# Heterogeneous graph neural networks for link prediction in biomedical networks

**DOI:** 10.1093/bioadv/vbaf187

**Published:** 2025-08-05

**Authors:** Junwei Hu, Michael Bewong, Selasi Kwashie, Wen Zhang, Hong-Yu Zhang, Zaiwen Feng

**Affiliations:** College of Informatics, Huazhong Agricultural University, Wuhan, Hubei, 430070, China; School of Computing, Mathematics & Engineering, Charles Sturt University, Wagga Wagga, NSW, 2678, Australia; Artificial Intelligence & Cyber Futures Institute, Charles Sturt University, Bathurst, NSW, 2795, Australia; Artificial Intelligence & Cyber Futures Institute, Charles Sturt University, Bathurst, NSW, 2795, Australia; College of Informatics, Huazhong Agricultural University, Wuhan, Hubei, 430070, China; Engineering Research Center of Agricultural Intelligent Technology, Ministry of Education, Wuhan, Hubei, 430070, China; Hubei Key Laboratory of Agricultural Bioinformatics, Huazhong Agricultural University, Wuhan, Hubei, 430070, China; College of Informatics, Huazhong Agricultural University, Wuhan, Hubei, 430070, China; Engineering Research Center of Agricultural Intelligent Technology, Ministry of Education, Wuhan, Hubei, 430070, China; Hubei Key Laboratory of Agricultural Bioinformatics, Huazhong Agricultural University, Wuhan, Hubei, 430070, China; College of Informatics, Huazhong Agricultural University, Wuhan, Hubei, 430070, China; Engineering Research Center of Agricultural Intelligent Technology, Ministry of Education, Wuhan, Hubei, 430070, China; Hubei Key Laboratory of Agricultural Bioinformatics, Huazhong Agricultural University, Wuhan, Hubei, 430070, China

## Abstract

**Summary:**

Heterogeneous graph neural networks (HGNNs) are gaining popularity as powerful tools for analysing complex networks with diverse node types often referred to as heterogeneous graphs. While existing HGNNs have been successfully used within the context of social and information networks, their application in biomedicine remains limited. In this study, we posit the utility of readily available generic HGNNs in addressing the link prediction tasks in biomedical settings. Thus, we conduct a benchmarking study of 42 techniques including nine generic HGNNs across eight biomedical datasets using several evaluation metrics. Our results show that the recently developed and readily available generic HGNNs achieve comparable and sometimes better results when compared with the specialized biomedical methods across all evaluation metrics. For instance, the generic HGNN *Simple-HGN* achieves the best results in four of the eight datasets and shows equivalent performance to the biomedical methods on the remaining datasets. Furthermore, this work analyses and presents useful guidelines to practitioners on how to optimally set complex hyperparameters which underpin the HGNNs.

**Availability and implementation:**

Finally, this work makes publicly available, via https://github.com/Zaiwen/Link_Prediction_in_Biomedical_Network, the benchmarking framework and source codes which underpin this study.

## 1 Introduction

The study of the relationships between biomedical entities (e.g. protein and drug interactions) has proven to be very significant, with several practical applications such as understanding disease mechanisms, aiding new drug developments and generating previously unknown insights on gene functions (Tsuyuzaki and Nikaido 2017). However, using wet-lab experiments in such studies is costly, risky, and often untenable. Instead, computer models, simulations, and computational methods tend to be faster, economical, and more accurate in determining potential relationships between biomedical entities.

Advances in bioinformatics research have facilitated the ability to generate and analyse complex biomedical data (Su *et al.* 2020). Graph representations (a.k.a. networks) are a powerful data structure widely used to represent biomedical entities (i.e. nodes) and their relationships (i.e. edges or links) (Yue *et al.* 2020). Indeed, in other domains, graph neural networks (GNNs) have been proposed for social network analysis, medical image processing, and biological information mining (cf. Perozzi *et al.* 2014, Tang *et al.* 2015, Grover and Leskovec 2016, Ribeiro *et al.* 2017). It is thus important to understand the merits of existing GNNs w.r.t. the four main biomedical link prediction tasks viz drug-drug interaction (DDI) prediction, drug-target interaction (DTI) prediction, protein-protein interaction (PPI) prediction, and drug-disease association (DDA) prediction. This will allow practitioners to determine the most appropriate schemes, and the conditions under which to use them. In Yue *et al.* (2020), the only other such benchmarking study, selected several representative graph embedding methods and performed systematic comparisons on node classification and link prediction tasks in biomedical networks. However, they focus on relatively simpler homogeneous networks with only one type of relationship. In most biomedical application scenarios, it is crucial to represent the heterogeneity of the different types of biomedical entities interacting with each other. Unlike the work in Yue *et al.* (2020), our study considers more realistic heterogeneous networks and analyses various heterogeneous network-based methods in completing link prediction tasks.

Various heterogeneous graph neural networks (HGNNs) (Wang *et al.* 2019, Yun *et al.* 2019, Yang *et al.* 2020a) have been proposed to solve relevant tasks including link prediction (Schlichtkrull *et al.* 2018, Zhang *et al.* 2019, Xu *et al.* 2021), node classification (Wang *et al.* 2019, 2021a, Fu *et al.* 2020), and knowledge-based recommendation (Zhao *et al.* 2017, Fan *et al.* 2019, Shi *et al.* 2019). However, they have primarily been evaluated on nonbiomedical networks like bibliographic networks, social networks, and e-commerce networks. More recently, a number of heterogeneous biomedical networks such as ProGO-Net (Kulmanov *et al.* 2021), DeepViral-Net (Liu-Wei *et al.* 2021), deepDR-Net (Zeng *et al.* 2019), NeoDTI-Net (Wan *et al.* 2019), etc. have been generated, making it possible, in this work, to assess the efficacy of heterogeneous graph representation techniques in addressing link prediction tasks more comprehensively.

It is worth noting that while purposefully designed heterogeneous network representation techniques for biomedical applications such as CDHGNN (Lu *et al.* 2023) exists, there is very little understanding of how relatively simpler generic HGNN techniques such as heterogeneous graph attention network (HAN) (Wang *et al.* 2019), metapath aggregated graph neural network (Fu *et al.* 2020), and heterogeneous graph transformer (HGT) (Hu *et al.* 2020) could be effectively adopted. This is important because generic HGNNs are not task-driven. Thus, unlike the techniques specifically designed for biomedical networks (i.e. well-known and established biomedical link prediction methods simply referred to as biomedical methods hereon in), the learned embeddings in generic HGNNs can also be viewed as complementary representations of biological features. In contrast, most existing biomedical methods are designed for specific tasks, limiting their applicability to broader problems. For instance, Li *et al.* (2023b) proposes a novel model called a pre-trained heterogeneous graph neural network model for drug-drug interaction prediction (HetDDI) that utilizes a pretrained heterogeneous graph neural network (HGNN) for DDI prediction by considering both the structure of drug molecules and the wealth of semantic information from biomedical knowledge graph. Similarly Li *et al.* (2023a) proposes a special type of GNN called metapath-aggregated heterogeneous graph neural network (MHGNN) and focuses on capturing more complex structures and richer semantic information within the biological data for DTI prediction. However, in both cases, HetDDI and MHGNN focus on specific biomedical link prediction tasks (i.e. DDI and DTI, respectively), and the representations derived for each task are not transferable to other tasks. Therefore, an effective and general heterogeneous graph computation method is necessary when practitioners want to handle more than one biomedical link prediction task. In addition, it can also serve as an auxiliary tool for studying and analysing biomedical networks, providing insights for designing specialized methods.

**Figure 1.**
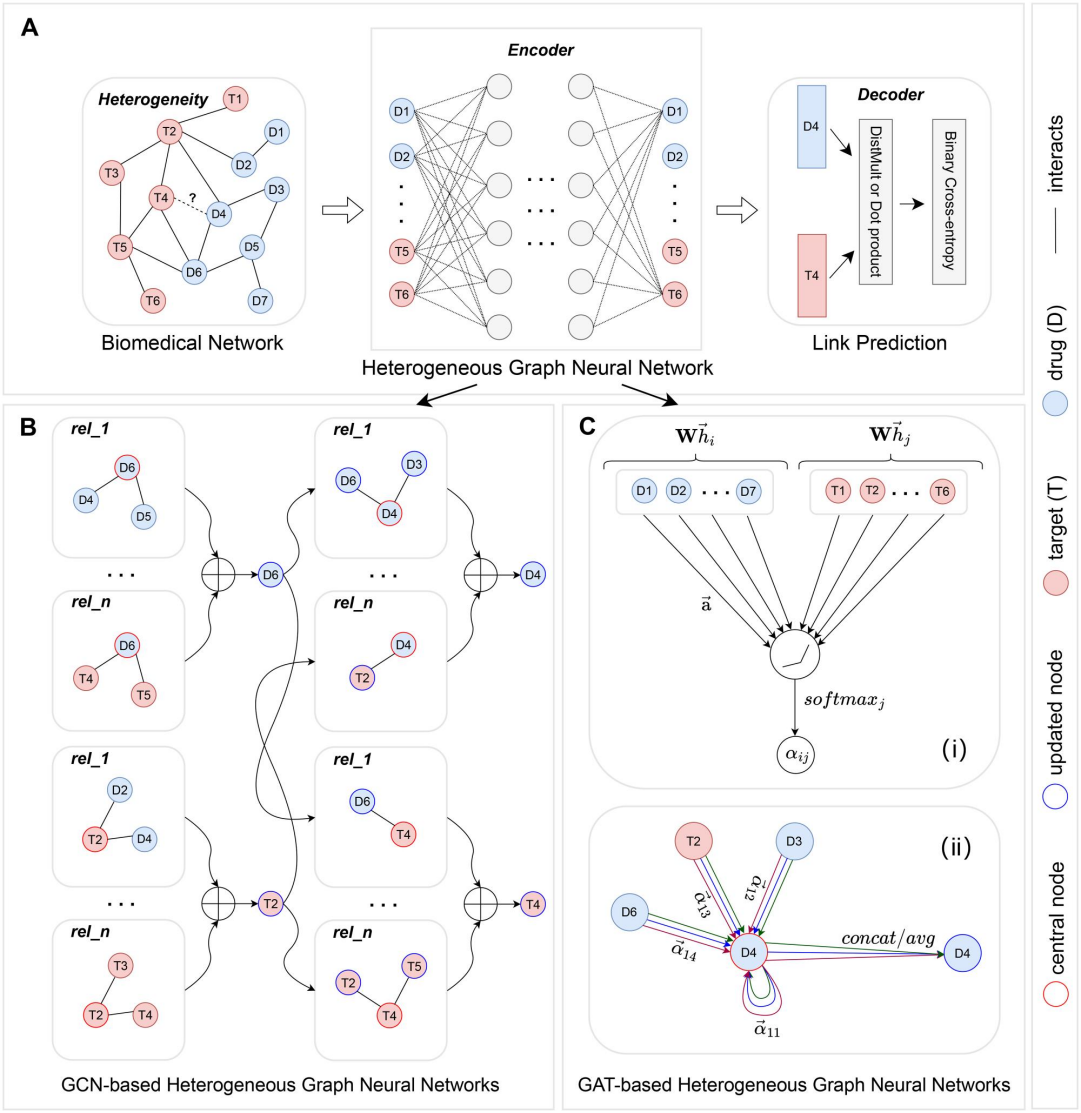
(A) Pipeline for applying heterogeneous graph neural networks for link prediction in biomedical networks. Step 1: Input a heterogeneous biomedical network. Step 2: Heterogeneous graph neural network is used as encoder to transform high-dimensional network into a low-dimensional node representation. Step 3: The obtained node representations are used as input features into a DistMult decoder or perform Dot product calculations to predict potential entity relationships. (B) GCN-based heterogeneous graph neural networks: Aggregation of neighbor features based on the weights corresponding to different relationship types (i.e. *rel_1*, ⋯,*rel_n*), and update of the feature vector of the central nodes (i.e. represented by nodes with red border) through a weighted summation calculation. The nodes with blue border denote an updated node/output. C. GAT-based Heterogeneous Graph Neural Networks: (i) Attention mechanism *a*(**W**${\vec{h}}_{i}$, **W**${\vec{h}}_{j}$ employed and parametrized by a weight vector $\vec{a}$, applying a LeakyReLU activation; (ii) Multi-head attention mechanism, with *K* = 3 heads, is illustrated focusing on a central node (i.e. represented by node with red border) and its neighborhood, and different arrow styles and colors distinguish independent attention calculations performed by each head. The features aggregated from each head are then concatenated or averaged to obtain the final output (i.e. represented by node with blue border).

In this work, we posit the utility of existing generic HGNNs in addressing biomedical link prediction tasks more effectively than the purpose-built biomedical methods. There are two main categories of generic HGNN methods namely, graph convolutional network-based heterogeneous graph neural networks (GCN-based HGNNs) and graph attention network-based heterogeneous graph neural networks (GAT-based HGNNs). Figure 1A illustrates the general workflow of applying various HGNNs to the task of link prediction, while Fig. 1B and C present an overview of GCN-based and GAT-based HGNNs, respectively. In the ensuing, we provide an overview of the recently developed generic HGNNs. Then, we benchmark these techniques in comparison with purposefully designed HGNN techniques across eight commonly used biomedical databases across four link prediction tasks: DDI, DTI, PPI, and DDA. The benchmarking demonstrates the potential of recently developed generic HGNN methods for these biomedical tasks. While recent studies (Hong *et al.* 2020, Ji *et al.* 2021, Yang *et al.* 2021) review the technical details of heterogeneous graph representation learning methods, this is the first work of its kind to conduct a systematic benchmarking of their performance in heterogeneous biomedical datasets. Specifically, we make the following contributions:

- We provide an overview of the recent generic HGNN methods and show that they are a competitive alternative for biomedical link prediction compared with the so-called biomedical methods such as Onto2Vec (Smaili *et al.* 2018), OPA2Vec (Smaili *et al.* 2019), NeoDTI (Wan *et al.* 2019), DTINet (Luo *et al.* 2017), deepDR (Zeng *et al.* 2019). In particular, the generic HGNNs perform markedly better on half of the experimental datasets across all metrics while showing comparable results on the remaining datasets.
- We conduct a comprehensive benchmarking study, the first of its kind, over 33 state-of-the-art methods using eight datasets across the four main biomedical link prediction tasks (i.e. DDA, DDI, PPI, DTI); and further systematically evaluate nine generic HGNNs against these link prediction tasks. In doing so, we collate the source codes of 42 different techniques in relation to biomedical link prediction as a resource in this article.
- We present useful guidelines, based on our findings, to researchers and practitioners on the influence of parameters on the various HGNN methods and how they should be determined to achieve effective results. Further, we make our benchmarking framework and datasets publicly available via https://github.com/Zaiwen/Link_Prediction_in_Biomedical_Network.

The rest of this work is organised as follows: In Section 2, we detail the heterogeneous graph based link prediction problem and present existing HGNNs. In Section 3, we introduce the whole pipeline for link prediction. We then present the experiments and analysis in Section 4 and a conclusion in Section 5.

## 2 Problem formulation

We formalize the heterogeneous graph representation learning problem, which was introduced in Cen *et al.* (2019) and Dong *et al.* (2017), and briefly describe the problem definition of link prediction.

Definition 1 (Heterogeneous Graph).

A heterogeneous graph can be formulated as a graph G=(V,E,ϕ,ψ), where each node v∈V is associated with a specific node type determined by the mapping function ϕ:V→O, and each edge e∈E is categorized into a distinct edge type defined by the mapping function ψ:E→R. Here, R represents the set of all edge types, and O denotes the set of all node types. If |R|+|O|>2, the network is heterogeneous; otherwise, it is homogeneous.

Figure 1A shows a heterogeneous graph representation of a simple biomedical network containing two entity types drug (D) and target (T) showing the interaction between drugs and targets, i.e. a DTI graph. The figure illustrates how a HGNN can be used to represent the relationships between the nodes (encoder) and predict the presence or absence of a previously unknown link between any two nodes of interest via the decoder.

Definition 2 (HGNN).

Given a heterogeneous graph G, HGNN aims to derive a representation vector $h_{v}^{\left(l\right)}\in R^{d_{l}}$ for each node v via l-layer transformations, utilizing both the graph structure and the initial node feature $h_{v}^{\left(0\right)}\in R^{d_{0}}$, where $d_{0}$ and $d_{l}$ represent the feature dimensionality of node v in layer 0 and l respectively. These learned representations are applicable to different downstream tasks such as knowledge-based recommendation, node classification, and link prediction.

We present a brief overview of different HGNNs, which can be categorized into two groups: GCN-based HGNNs and GAT-based HGNNs. Table 1, provides a summary of various HGNN methods and their implementations.

**Table 1.** Summary of existing GCN-based and GAT-based methods.

Category	Method	Source code	Library	Year	Reference
GCN-based HGNNs	R-GCN	https://github.com/tkipf/relational-gcn	Keras	2018	(Schlichtkrull *et al.* 2018)
	CompGCN	https://github.com/malllabiisc/CompGCN	PyTorch	2020	(Vashishth *et al.* 2019)
GAT-based HGNNs	R-GAT	https://github.com/babylonhealth/rgat	Tensorflow	2019	(Busbridge *et al.* 2019)
	HAN	https://github.com/Jhy1993/HAN	Tensorflow	2020	(Wang *et al.* 2019)
	HPN	https://github.com/BUPT-GAMMA/OpenHGNN	PyTorch	2021	(Ji *et al.* 2021)
	ie-HGCN	https://github.com/kepsail/ie-HGCN/	PyTorch	2021	(Yang *et al.* 2021)
	HGT	https://github.com/acbull/pyHGT	PyTorch	2019	(Hu *et al.* 2020)
	HetSANN	https://github.com/didi/hetsann	Tensorflow	2020	(Hong *et al.* 2020)
	Simple-HGN	https://github.com/THUDM/HGB	PyTorch	2021	(Lv *et al.* 2021)

GCN-based HGNNs: GCN (Kipf and Welling 2016a) is a pioneering GNN model which considers the features of neighboring nodes and transforms them to update the central node feature. This process essentially captures the graph structure within the calculations involving node features. The lth layer of a GCN is defined as:

(1) $$H^{\left(l\right)}=\sigma (\hat{A}H^{\left(l-1\right)}W^{\left(l-1\right)})$$

where $H^{\left(l\right)}$ represents the updated representations for all nodes after processing by the lth layer, σ is the activation function, $\hat{A}$ denotes the normalized adjacency matrix with self-connections, and $W^{\left(l-1\right)}$ represents a trainable weight matrix. Figure 1B illustrates a GCN. The figure only shows two layers of updates where in each layer all nodes are updated via their feature representations by aggregating their neighbor nodes. We use D6 (drug node) and T2 (target node) to illustrate this update in the first layer and D4 (drug node) and T4 (target node) to illustrate this update in the second layer. The number of layers is determined by the user. With the emergence of heterogeneous networks, many researchers have extended GCN to enable it to work better in heterogeneous graphs as follows.

- R-GCN (Schlichtkrull *et al.* 2018). Relational graph convolutional network (R-GCN) is an extension of GCN designed for relational graphs (containing multiple edge types). It performs convolution by weighted summation of the outputs of multiple ordinary graph convolutions. Each convolution considers specific types of edges, enabling the model to capture richer information from the network structure. For each node *i*, the lth layer of convolution in R-GCN is defined as:
  (2) $$h_{i}^{\left(l\right)}=\sigma \left(\sum_{r\in R}\sum_{j\in N_{i}^{r}}\frac{1}{c_{i,r}}W_{r}^{\left(l-1\right)}h_{j}^{\left(l-1\right)}+W_{0}^{\left(l-1\right)}h_{i}^{\left(l-1\right)}\right)$$
  where $h_{i}^{\left(l\right)}$ is the representation of node *i* after lth layer, $N_{i}^{r}$ represents the collection of indices for all neighboring nodes of node *i* corresponding to relation r∈R, and $c_{i,r}$ denotes a normalization constant.
- CompGCN (Vashishth *et al.* 2019). CompGCN expands upon R-GCN by utilizing diverse entity-relation composition operations, thereby embedding nodes and relations jointly.
  GAT-based HGNNs: Graph attention network (GAT) (Veličković *et al.* 2017) is similar to GCN, using the attention mechanism to update the feature of the central node by aggregating neighbor features. In comparison with GCN, the GAT substitutes the average aggregation from neighbors, denoted by $\hat{A}H^{\left(l-1\right)}$, with a weighted aggregation. Followed by this approach, the weight $\alpha_{ij}$ for each edge 〈i,j〉 is determined by an attention mechanism:
  (3) $$\alpha_{ij}=\frac{\;\text{exp}(\text{LeakyReLU}(a^{T}[Wh_{i}||Wh_{j}]))}{\Sigma_{k\in N_{i}}\;\text{exp}(\text{LeakyReLU}(a^{T}[Wh_{i}||Wh_{k}]))}$$
  where ***a*** and ***W*** denote learnable weights, ‖ is the concatenation operator, and $N_{i}$ represents the neighbors of node *i*. It is worth noting that the layer *l* is omitted here for simplicity. Additionally, the multihead attention technique (Vaswani *et al.* 2017) can be incorporated to enhance performance. Figure 1C illustrates a GAT. As an example, node D4 first learns the attention weight between its connected nodes (i.e. D6, T2, and D3) through the activation function *softmax*, and then updates its representation through the weighted aggregation. For heterogeneous graphs, most work extends the original attention mechanism to calculate the attention coefficient based on the relationships between different entities. These works are proven to show encouraging results in complex network analysis tasks, and are described as follows.
- R-GAT (Busbridge *et al.* 2019). Relational graph attention network (R-GAT) extends attention mechanisms to the relational graph through calculation weight $\alpha_{ij}$ for each edge 〈i,j〉:
  (4) $$\alpha_{ij}^{r}=\frac{\;\text{exp}(\sigma (a^{T}[W_{r}h_{i}||W_{r}h_{j}]))}{\Sigma_{r\in R}\Sigma_{k\in N_{i}^{r}}\;\text{exp}(\sigma (a^{T}[W_{r}h_{i}||W_{r}h_{k}]))}$$
  where $W_{r}$ represents the learnable parameters of a shared linear transformation. Then, the attention mechanism of Equation (4) is combined with the neighborhood aggregation step (Schlichtkrull *et al.* 2018) to obtain:
  (5) $$h_{i}^{\left(l\right)}=\sigma \left(\sum_{r\in R}\sum_{j\in N_{i}^{r}}\alpha_{ij}^{r}W_{r}h_{j}^{\left(l-1\right)}\right)$$
- HAN (Wang *et al.* 2019). HAN employs meta-paths instead of focusing on one-hop neighbors to capture higher-order proximity. For a given meta-path *M*, the representation of node *u* is derived from the aggregation of its neighbors based on meta-path, i.e. $N_{M}(u)=\{u\}\cup \{v|v\;\text{connects}\;\text{with}\;u\;\text{via}\;\text{the}\;\text{meta-path}\;M\}$. HAN introduces an attention mechanism aimed at learning different weights to these neighbors, highlighting the more important ones for understanding node *u*:
  (6) $$\alpha_{uv}^{M}=\frac{\;\text{exp}(\sigma (a_{M}^{T}[h_{u}^{\prime }||h_{v}^{\prime }]))}{\Sigma_{k\in N_{M}(u)}\;\text{exp}(\sigma (a_{M}^{T}[h_{u}^{\prime }||h_{k}^{\prime }]))}$$
   
  (7) $$h_{u}^{M}=\sigma \left(\sum_{v\in N_{M}(u)}\alpha_{uv}^{M}h_{v}^{\prime }\right)$$
  where $h_{u}^{\prime }$ and $h_{v}^{\prime }$ are the projected feature vectors of node *u* and *v*, respectively, $a_{M}$ represents the node-level attention vector of the meta-path *M*. Based on the embedding $h_{u}^{M}$ obtained from specific meta-path, HAN assigns weights to different meta-paths through the calculation of semantic-level attention:
  (8) $$\beta_{M}=\frac{\;\text{exp}(\frac{1}{\left|V\right|}\Sigma_{u\in V}\;q^{T}\tanh(Wh_{u}^{M}+b))}{\Sigma_{M^{\prime }}\;\text{exp}(\frac{1}{\left|V\right|}\Sigma_{u\in V}\;q^{T}\tanh(Wh_{u}^{M^{\prime }}+b))}$$
   
  (9) $$e_{u}=\sum_{M}\beta_{M}h_{u}^{M}$$
  where ***W*** denotes the weight matrix, ***b*** represents the bias vector, ***q*** refers to the semantic-level attention vector, and $e_{u}$ is the final representation of node *u*.
- HPN (Ji *et al.* 2021). Heterogeneous graph propagation network (HPN) extends HAN model by incorporating two essential mechanisms: semantic propagation and semantic fusion. The former mechanism emphasizes the local semantics of each node during the process of aggregating from its neighbors and alleviates semantic confusion at the node level. Meanwhile, the latter mechanism learns the relative importance of different meta-paths for the specific task, and generates an optimally weighted combination of semantic-specific node embeddings.
- HGT (Hu *et al.* 2020). Inspired by the Transformer structure (Vaswani *et al.* 2017, Devlin *et al.* 2019), HGT utilizes individual edge types to parameterize the attention mechanism resembling that of Transformer. In particular, HGT maps *v* into a *Query* vector and *u* into a *Key* vector for each edge 〈u,v〉, subsequently computing their dot product to derive attention.
  (10) $$Q_{v}^{i}=Q_{\phi (v)}^{i}(h_{v}^{\left(l\right)}),\;K_{u}^{i}=K_{\phi (u)}^{i}(h_{u}^{\left(l\right)})$$
   
  (11) $$ATT_{\mathrm{head}}^{i}(u,v)=\left(K_{u}^{i}W_{\psi_{(}u,v)}^{\mathrm{ATT}}Q_{v}^{i\;T}\right)\frac{\mu (\phi (u),\psi (u,v),\phi (v))}{\sqrt{d}}$$
   
  (12) $${\text{Attention}}_{\mathrm{HGT}}(u,v)=\underset{u\in N(v)}{\text{softmax}}\left(\underset{i\in [1,k]}{\left|\right|}{\text{ATT}}_{\text{head}}^{i}(u,v)\right)$$
  where $h_{v}^{\left(l\right)}$ and $h_{u}^{\left(l\right)}$ are the output of the $l^{th}$ HGT layer, $Q_{\phi (v)}^{i}$ and $K_{\phi (u)}^{i}$ represent the node type-aware linear projection function, ${\text{ATT}}_{\text{head}}^{i}$ denote the ith attention head, $W_{\psi_{(}u,v)}^{\mathrm{ATT}}$ is an edge-based matrix, μ is a prior tensor representing adaptive scaling to the attention, and *k* represents the number of attention heads.
- HetSANN (Hong *et al.* 2020). Heterogeneous Graph Structural Attention Neural Network (HetSANN), similar to HGT, utilizes an attention mechanism to analyse the importance of different types of nodes around a specific query node, enabling it to capture both interactions of intertype nodes and assign different weights to neighbors during message aggregation.
- Simple-HGN (Lv *et al.* 2021). Lv *et al.* were inspired by the effectiveness of the straightforward GAT over more complex and specialized HGNNs, leading them to propose Simple-HGN. This approach extends the original graph attention mechanism by incorporating edge type information when calculating attention scores between nodes. To be specific, Simple-HGN allocates a *d*-dimensional embedding $h_{r}$ for each type of edge r=ψ(e)∈R in each layer. It then employs both edge type embeddings and node embeddings to compute the attention score:
  (13) $$\alpha_{ij}=\frac{\;\text{exp}(\sigma (a^{T}[Wh_{i}||Wh_{j}||W_{r}h_{\psi (\langle i,j\rangle )}]))}{\sum_{r\in R}\sum_{k\in N_{i}^{r}}\;\text{exp}\;(\sigma (a^{T}[Wh_{i}||Wh_{k}||W_{r}h_{\psi (\langle i,k\rangle )}]))}$$
  where $W_{r}$ represents a learnable matrix to transform type embeddings, and ψ(〈i,j〉) denotes the type of edge 〈i,j〉.
- ie-HGCN (Yang *et al.* 2021). Interpretable and Efficient Heterogeneous Graph Convolutional Network (ie-HGCN) is crafted to learn embeddings for heterogeneous graphs through a GCN that is specialized for node types. Initially, ie-HGCN maps the representations of diverse types of neighboring nodes into a unified semantic space. Then, in node-level aggregation, it treats the heterogeneous graph as a set of several bipartite graphs and applies GCN for bipartite graphs. Finally, ie-HGCN utilizes the attention mechanism to aggregate diverse types of neighboring nodes to generate node embeddings in type-level aggregation.

There are generally more GAT-based methods compared with GCN-based methods because GAT-based methods are often seen to perform better in complex networks (Yang *et al.* 2020b; Karagiannakos 2021). This is in part due the ability to adapt relationship weights of edges between nodes during the learning process of GAT, unlike GCN. More details of these models (cf. Table 1) are described in Supplementary Section S1, available as supplementary data at *Bioinformatics Advances* online.

Definition 3 (Link prediction).

Following Lü and Zhou (2011), the task of link prediction in biomedicine is to leverage existing relationships between known biological entities to identify new/potential interactions that might exist between them.

## 3 Link prediction

The aim of the link prediction task is to estimate a previously unknown relationship between entities in the biomedical network. For example, one might be interested in determining the relationship between the drug node (D4) and the target node (T4) in Fig. 1A. In Fig. 1A, first, the heterogeneous biomedical network with network structure information is the input into the HGNN (as an encoder) to learn the low-dimensional embedding representation of entities in the vector space. Subsequently, Fig. 1B and C show two different types of HGNNs, namely GCN-based HGNNs and GAT-based HGNNs, which aggregate the neighbor node features of the central node in different ways to update the feature representation of the central node. Finally, the learned feature representations such as D4 and T4 will be input into the decoder to determine whether they have potential links.

We utilize two decoders, *DistMult* (Yang *et al.* 2014) and Dot product, to predict the new interactions between entities. For an entity pair *u*, *v*, and a target edge type *r*, *DistMult* determines whether they are linked through the following formula:

(14) $${\text{Prob}}_{r}(u,v)=\text{sigmoid}\left(\text{HGNN}{\left(u\right)}^{T}M_{r}\text{HGNN}(v)\right)$$

where $M_{r}$ is a learnable square matrix for relational type r∈R. Similarly, Dot product directly performs $\text{HGNN}{\left(u\right)}^{T}\text{HGNN}(v)$ to identify whether *u*, *v* exist a link with relationship type *r*. We reported the best results from Dot product or *DistMult* decoders, optimized using binary cross-entropy.

## 4 Experiments

Our experimental goal is to evaluate the effectiveness of general purpose HGNNs for biomedical networks and, among other things, compare their performance to the so-called state-of-the-art biomedical methods. Finally, we provide valuable guidelines for practitioners by conducting grid searches to identify optimal hyperparameter settings for each HGNN method. In this section, we first introduce the eight datasets used in this study for link prediction tasks, and then comprehensively compare a total of 42 techniques including nine selected HGNN techniques on these datasets (cf. Section 2).

### 4.1 Experimental settings and evaluations

We utilize eight biomedical networks in this work, namely DeepViral-Net (Liu-Wei *et al.* 2021), ProGO-Net (Kulmanov *et al.* 2021), NeoDTI-Net (Wan *et al.* 2019), deepDR-Net (Zeng *et al.* 2019), CTD-DDA (Yue *et al.* 2020), NDFRT-DDA (Yue *et al.* 2020), DrugBank-DDI (Yue *et al.* 2020), and STRING-PPI (Yue *et al.* 2020). The details of eight datasets are provided in Supplementary Section S2, available as supplementary data at *Bioinformatics Advances* online. We identified 33 state-of-the-art methods (cf. Table 2) for benchmarking on the eight datasets across the four main link prediction tasks. For each task, we identify key evaluation metrics from the literature (Wan *et al.* 2019, Zeng *et al.* 2019, Yue *et al.* 2020, Kulmanov *et al.* 2021, Liu-Wei *et al.* 2021, Wang *et al.* 2021b), as shown in Supplementary Section S4, available as supplementary data at *Bioinformatics Advances* online. We use OpenHGNN (Han *et al.* 2022), an open-source Python package for heterogeneous graph representation learning, to learn low-dimensional vector representations for recent HGNNs as shown in the Table 1. It is worth noting that these eight datasets and baseline methods are designed for handling different link prediction scenarios. For a fair comparison, we ensure that amongst other experiments, we conduct the same link prediction tasks that were conducted in the baseline methods. The settings and prediction tasks on two special datasets are described as follows, and more details on the experimental settings are described in Supplementary Section S5, available as supplementary data at *Bioinformatics Advances* online.

**Table 2.** The 33 state-of-the-art baseline methods.^a^

Model	Reference	Model	Reference	Model	Reference
BioERP	(Wang *et al.* 2021b)	MSCMF	(Zheng *et al.* 2013)	Katz	(Singh-Blom *et al.* 2013)
DL2Vec	(Chen *et al.* 2021)	HNM	(Wang *et al.* 2014)	Laplacian	(Belkin and Niyogi 2003)
Onto2Vec	(Smaili *et al.* 2018)	DTINet	(Luo *et al.* 2017)	GF	(Ahmed *et al.* 2013)
OPA2Vec	(Smaili *et al.* 2019)	BLMNII	(Mei *et al.* 2013)	SVD	(Kalman 1996)
Node2Vec	(Grover and Leskovec 2016)	DT-Hybrid	(Alaimo *et al.* 2013)	HOPE	(Ou *et al.* 2016)
EL Embeddings	(Kulmanov *et al.* 2019)	NetLapRLS	(Xia *et al.* 2010)	Grarep	(Cao *et al.* 2015)
TransE	(Bordes *et al.* 2013)	deepDR	(Zeng *et al.* 2019)	DeepWalk	(Perozzi *et al.* 2014)
SimResnik	(Resnik 1995)	KBMF	(Gönen *et al.* 2013)	Struc2Vec	(Ribeiro *et al.* 2017)
SimLin	(Lin *et al.* 1998)	SVM	(Cortes and Vapnik 1995)	LINE	(Tang *et al.* 2015)
SiameseNN	(Kulmanov *et al.* 2021)	RF	(Breiman 2001)	SDNE	(Wang *et al.* 2016)
NeoDTI	(Wan *et al.* 2019)	RWR	(Cao *et al.* 2014)	GAE	(Kipf and Welling 2016b)

a We summarize baseline models’ description and relevant source code links in Supplementary Section S3, available as supplementary data at *Bioinformatics Advances* online.

- **DeepViral-Net:** Following the guidelines in Liu-Wei *et al.* (2021), we utilize the leave-one-family-out cross-validation strategy to test how well the model performs. We leave out the virus family 11 308 for testing, the results are separated into the full dataset and high confidence dataset where we filter PPIs with a MIscore (Villaveces *et al.* 2015) less than 0.4 in HPIDB (Ammari *et al.* 2016).
- **ProGO-Net:** Following the guidance in Kulmanov *et al.* (2021), the dataset randomly splits known protein-protein pairs into an 80% training set and 20% testing set, using 20% of the training set as a validation set. We utilize a prediction function, also used in TransE (Bordes *et al.* 2013), to rank all protein pairs that appear in the testing set to evaluate the model by Hit@10/100, mean rank (MR). The results are divided into Raw and Filtered categories in Kulmanov *et al.* (2021). Raw results consider all pairs of proteins in the testing set, whereas Filtered results exclude pairs found in the training or validation sets.

The interested reader, is also referred to the original articles of the baseline methods referenced in Table 2 for further details.

### 4.2 Link prediction results

#### 4.2.1 Comparison with recent HGNNs

In this section, we aim to evaluate the suitability of existing generic HGNNs for biomedical link prediction tasks. We conducted link prediction tasks on the eight biomedical networks (cf. Supplementary Section S2, available as supplementary data at *Bioinformatics Advances* online). Table 3 shows the overall performance of different generic HGNN representation learning methods. Note that the DeepViral-Net dataset displays the results on the full dataset, while the ProGO-Net dataset displays the Raw results (cf. Section 4.1).

**Table 3.** Performance of recent HGNNs on eight biomedical networks.^a^

Methods	DeepViral-Net		ProGO-Net		NeoDTI-Net		deepDR-Net		CTD-DDA		NDFRT-DDA		DrugBank-DDI		STRING-PPI	
	AUC	AUPR	AUC	AUPR	AUC	AUPR	AUC	AUPR	AUC	AUPR	AUC	AUPR	AUC	AUPR	AUC	AUPR
R-GCN	0.8847	0.8769	0.9364	0.9289	0.9213	0.9301	0.9415	0.9323	0.8915	0.8801	0.9197	0.9039	0.8042	0.7637	0.9413	0.9374
R-GAT	0.8973	0.8872	0.9509	0.9450	0.9107	0.9251	0.9422	0.9348	0.9010	0.8916	0.9682	0.9631	0.8350	0.7954	0.9534	0.9462
HAN	0.8659	0.8448	0.8884	0.8837	0.9555	0.9595	0.8942	0.8853	0.8633	0.8607	0.9117	0.8950	0.7786	0.7438	0.8816	0.8680
CompGCN	0.9228	0.9181	0.9613	0.9575	0.9135	0.9293	0.9380	0.9289	0.9034	0.8985	0.9585	0.9553	0.8670	0.8232	0.9697	0.9660
HPN	0.8812	0.8725	0.8396	0.8083	0.9343	0.9367	0.7874	0.7617	0.8430	0.8253	0.8135	0.7796	0.7746	0.7385	0.8142	0.7731
ie-HGCN	0.8710	0.8598	0.9633	0.9638	**0.9668**	**0.9720**	0.9573	0.9574	0.9195	0.9183	0.9654	0.9659	0.8822	0.8358	0.9603	0.9600
HGT	0.9145	0.9084	0.9672	0.9654	0.9166	0.9186	0.8856	0.8759	0.9357	0.9334	0.9618	0.9502	0.9007	0.8638	0.9642	0.9654
Simple-HGN	**0.9247**	**0.9191**	**0.9788**	**0.9769**	0.9102	0.9248	**0.9639**	**0.9608**	**0.9467**	**0.9430**	**0.9768**	**0.9704**	**0.9080**	**0.8770**	**0.9722**	**0.9723**
HetSANN	0.7792	0.8339	0.8211	0.8598	0.8586	0.8873	0.8171	0.8436	0.8305	0.8565	0.9359	0.9447	0.8224	0.7874	0.8874	0.9037

a The best results are marked in boldface. Additional experiments on other complex datasets can also be found in the Supplementary Section S10, available as supplementary data at *Bioinformatics Advances* online.

Generally, it can be observed that the recently proposed generic HGNNs perform well in biomedical network link prediction tasks. For example, the simple-HGN approach has shown excellent performance on almost all datasets, achieving the best results on multiple datasets. Further, the ie-HGCN method has also shown encouraging results on the NeoDTI-Net network, and all HGNN techniques performed over 75% on AUC and AUPR metrics across all biomedical networks. We observe that if the graph structure is relatively simple (e.g. CTD-DDA) and the difference between the importance of neighboring nodes to the central node is not significant, GCN-based HGNNs can be chosen. If the graph structure is complex (e.g. NeoDTI-Net) and there is a significant difference in the importance of neighboring nodes to the central node, GAT-based HGNNs can be chosen. Overall, GAT-based HGNNs is a more flexible and powerful model compared with GCN-based HGNNs.

To demonstrate their potential, we further validate their performance by comparing them with so-called state-of-the-art biomedical methods in the following section.

#### 4.2.2 Comparison with state-of-the-art methods

This section compares the top 3 generic HGNN techniques observed for each respective dataset from the previous section with the so-called biomedical methods.

Table 4 shows the results of novel virus-host protein interactions on full dataset and high confidence dataset of the DeepViral-Net data. CompGCN, HGT, and Simple-HGN, which are the generic HGNNs, generally perform much better than DL2Vec and BioERP methods, which are biomedical methods for the PPI prediction task in the DeepViral-Net data, on full dataset. However, on high confidence dataset, BioERP has a slight edge over Simple-HGN only in ACC value.

**Table 4.** Performance of recent HGNNs and baseline methods in DeepViral-Net.^a^

Methods	Full dataset			High confidence dataset		
	AUC	AUPR	ACC	AUC	AUPR	ACC
BioERP	0.881	0.803	0.796	0.854	0.771	**0.769**
DL2Vec	0.851	0.735	0.566	0.841	0.748	0.586
CompGCN	0.923	0.918	0.837	0.812	0.810	0.756
HGT	0.915	0.908	**0.842**	0.849	0.851	0.745
Simple-HGN	**0.925**	**0.919**	0.841	**0.855**	**0.863**	0.764

a The best results are marked in boldface.

Table 5 shows the human PPI prediction results in Raw and Filtered for the ProGO-Net data. From the comparison of the 10 baseline methods, Simple-HGN is superior to all the other state-of-the-art techniques across all evaluation metrics. Also, ie-HGCN and HGT show great results in MR value (i.e. better than baseline methods).

**Table 5.** Performance of recent HGNNs and baseline methods in ProGO-Net.^a^

Methods	Raw			Filtered		
	Hits@10	Hits@100	MR	Hits@10	Hits@100	MR
TransE	0.05	0.24	3960.4	0.11	0.29	3890.6
SimResnik	0.05	0.25	1933.6	0.09	0.30	1864.4
SimLin	0.04	0.20	2287.9	0.08	0.23	2218.7
SiameseNN	0.05	0.41	1881.1	0.15	0.64	1808.8
SiameseNN(ont)	0.05	0.38	1838.3	0.13	0.59	1766.3
EL Embeddings	0.01	0.22	1679.7	0.02	0.26	1637.7
Onto2Vec	0.05	0.24	2434.6	0.08	0.31	2391.2
OPA2Vec	0.03	0.23	1809.7	0.07	0.26	1767.6
DeepWalk	0.04	0.28	1942.6	0.10	0.34	1958.6
Node2Vec	0.03	0.22	1860.5	0.07	0.28	1813.1
ie-HGCN	0.05	0.36	1174.8	0.12	0.52	1109.7
HGT	0.05	0.34	957.7	0.10	0.49	892.0
Simple-HGN	**0.08**	**0.46**	**936.1**	**0.18**	**0.66**	**870.7**

a Other evaluation metrics can be found in Supplementary Section S6, available as supplementary data at *Bioinformatics Advances*. The best results are marked in boldface.

**Figure 2.**
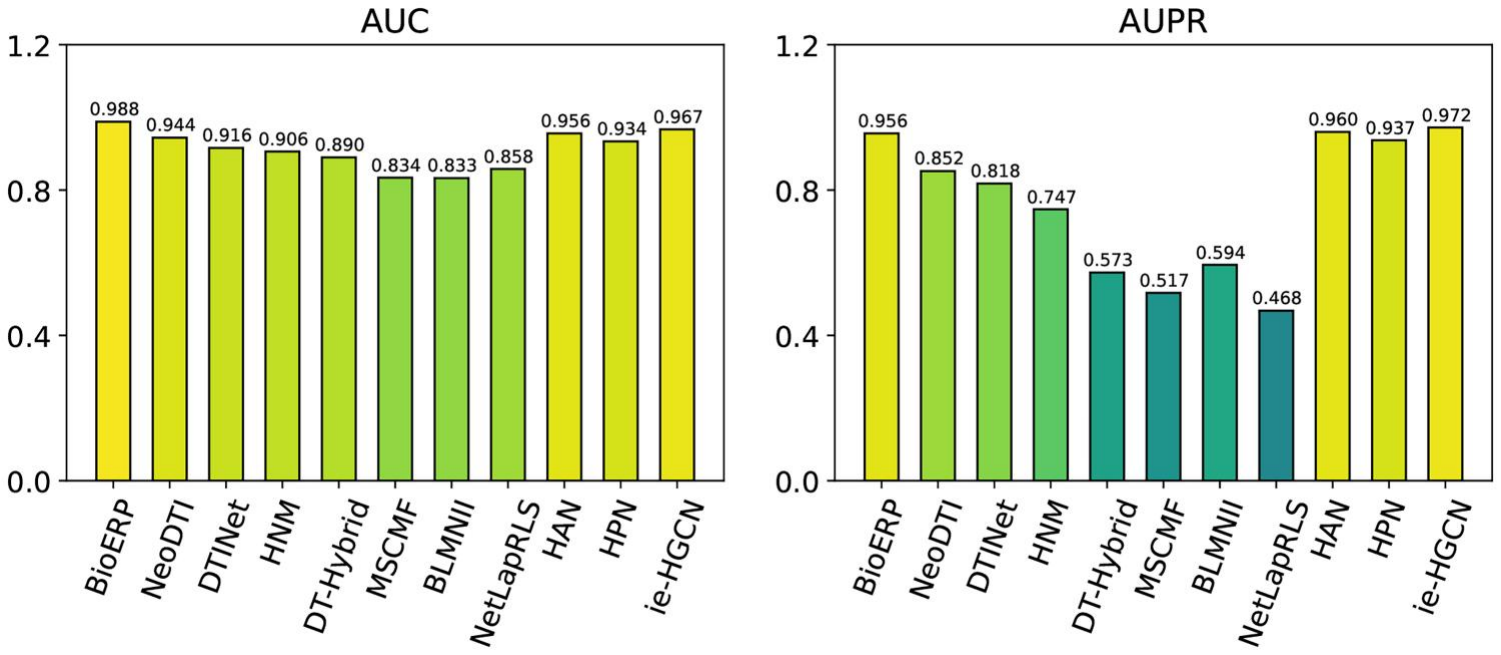
Result of recent HGNNs and baseline methods in NeoDTI-Net.

Figure 2 presents the performances of DTI predictions for the NeoDTI-Net dataset. Observing BioERP, purposefully designed for biomedical prediction tasks, shows better AUC results (i.e. 0.988), closely followed by ie-HGCN (i.e. 0.967). However, in terms of AUPR values, ie-HGCN performs better (i.e. 0.972) closely followed by HAN, another generic HGNN which performs better than NeoDTI, a method specifically designed for NeoDTI-Net. This further demonstrates that the recent generic HGNNs have great potential in biomedical tasks.

**Figure 3.**
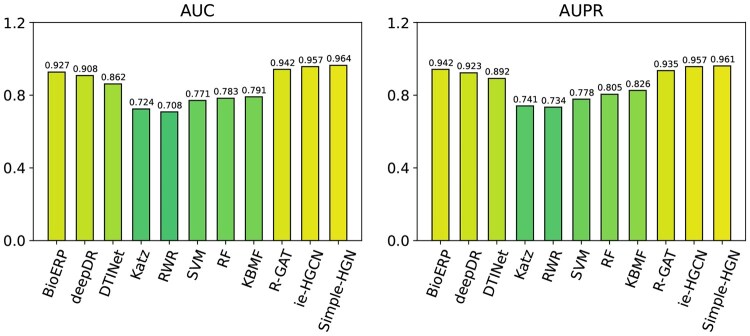
Result of recent HGNNs and baseline methods in deepDR-Net.

Figure 3 presents the DDA prediction results for deepDR-Net dataset, Simple-HGN, followed by ie-HGCN, both generic HGNN methods, are generally superior to all baseline methods, as evidenced by the higher AUC and AUPR values. Further, R-GAT, a generic HGNN method, has competitive performance with deepDR, designed specifically for deepDR-Net and the state-of-the-art method BioERP.

Table 6 shows the results of recent generic HGNNs and baseline methods on the four datasets, which consider only one relationship type, CTD-DDA, NDFRT-DDA, DrugBank-DDI, and STRING-PPI. We compare 12 baseline methods in terms of AUC and F1 values. First, it can be seen that ie-HGCN, HGT, and Simple-HGN demonstrate comparable performance compared with other 12 baseline methods. Second, these HGNNs may not necessarily perform very well in simple biomedical networks (i.e. NDFRT-DDA and DrugBank-DDI), however, in the STRING-PPI dataset, Simple-HGN performed significantly better than all other methods, with higher AUC value (≈4% increase), and higher F1 score (≈6% increase) compared with BioERP. Third, due to the sparse structure and simple semantics of single networks (e.g. CTD-DDA network), the general purpose graph embedding methods such as GraRep, Struc2Vec, LINE, etc. seem to be able to represent biomedical entities well, and outperform complex HGNNs in DDA and DDI prediction task.

**Table 6.** Performance of recent HGNNs and baseline methods in four networks.^a^

Method	CTD-DDA		NDFRT-DDA		DrugBank-DDI		STRING-PPI	
	AUC	F1	AUC	F1	AUC	F1	AUC	F1
BioERP	**0.966**	**0.907**	0.971	**0.939**	**0.930**	0.858	0.926	0.858
Laplacian	0.856	0.802	0.930	0.921	0.796	0.729	0.639	0.586
SVD	0.936	0.854	0.779	0.700	0.919	0.837	0.867	0.790
GF	0.884	0.805	0.720	0.655	0.882	0.802	0.810	0.747
HOPE	0.951	0.887	0.949	0.931	0.923	0.846	0.839	0.764
GraRep	0.960	0.900	0.963	0.934	0.925	0.846	0.894	0.822
DeepWalk	0.929	0.864	0.783	0.709	0.921	0.842	0.884	0.814
Node2Vec	0.911	0.835	0.819	0.741	0.902	0.819	0.828	0.756
Struc2Vec	0.965	0.903	0.958	0.921	0.904	0.830	0.909	0.841
LINE	0.965	0.904	0.962	0.935	0.905	0.829	0.859	0.795
SDNE	0.935	0.861	0.944	0.897	0.911	0.838	0.884	0.814
GAE	0.937	0.856	0.813	0.730	0.917	0.840	0.900	0.829
ie-HGCN	0.920	0.849	0.965	0.907	0.882	0.840	0.960	0.912
HGT	0.936	0.860	0.962	0.864	0.901	0.855	0.964	0.920
Simple-HGN	0.947	0.868	**0.977**	0.903	0.908	**0.863**	**0.972**	**0.926**

a Other evaluation metrics can be found in Supplementary Section S7, available as supplementary data at *Bioinformatics Advances* online. The best results are marked in boldface.

### 4.3 Influence of hyperparameters

In this section, we conduct further analyse to determine the influence of hyperparameters on the generic HGNNs. Hyperparameters are crucial for the predictive performance of machine learning models. Nonetheless, choosing appropriate hyperparameters is often challenging and can be time-consuming. Using a grid search approach, first, we investigate the impact of several critical common hyperparameters such as number of embedding dimensions and the GNN layers in various HGNN methods. Second, we analyse model specific hyperparameters that influence the performance of individual HGNN techniques. The results presented in this section can serve as general guidelines for practitioners to enable effective setting of hyperparameters to achieve better performance.

#### 4.3.1 Effect of embedding dimensions

**Figure 4.**
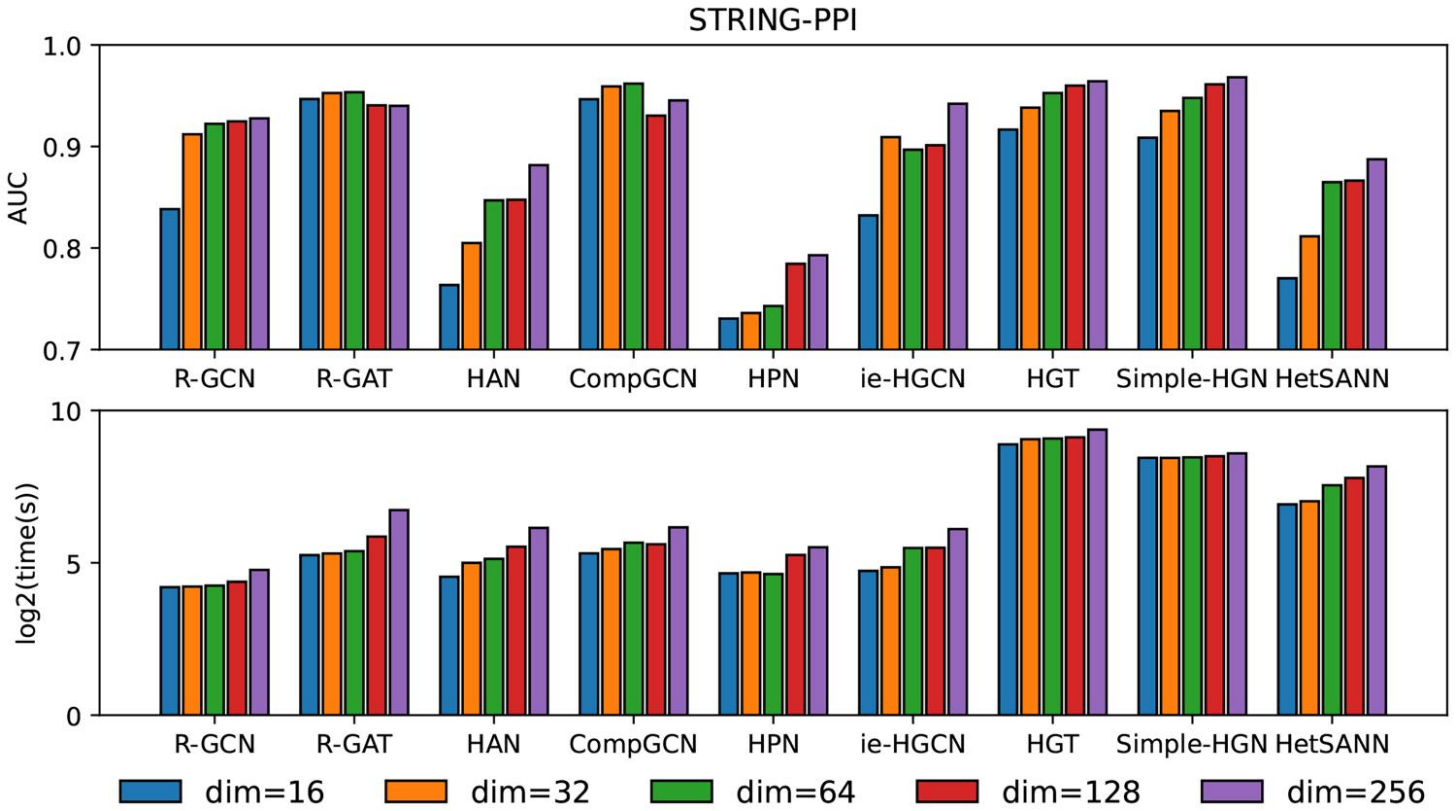
Performance and training time of different HGNNs in STRING-PPI under dimensionality settings.

The embedding dimension usually directly affects the performance of model prediction because it contains the structure and feature information of the graph. We evaluate how different embedding dimensions (i.e. 16, 32, 64, 128, 256) affect predictive performance and time efficiency of HGNNs. Figure 4 shows the predictive performance and time efficiency under different embedding dimensionalities for the STRING-PPI dataset. Intuitively, increasing the HGNN embedding dimension can lead to better predictive performance, because higher dimensions have the capacity to encode more valuable information. The performance of R-GAT and CompGCN tends to saturate at 64 dimensions, while other techniques achieve the highest performance at 256 dimensions. In terms of full training time cost (in seconds), for embedding dimensions below 64, it initially gradually increases, but when the dimension rises above 64, it often increases sharply. Thus, it seems the ideal dimensionality for HGNN methods in biomedical link prediction tasks is around 64 when taking into account both performance and time efficiency. The influence of dimensionality settings on other datasets in Supplementary Section S8, available as supplementary data at *Bioinformatics Advances* online.

#### 4.3.2 Effect of GNN layers

**Figure 5.**
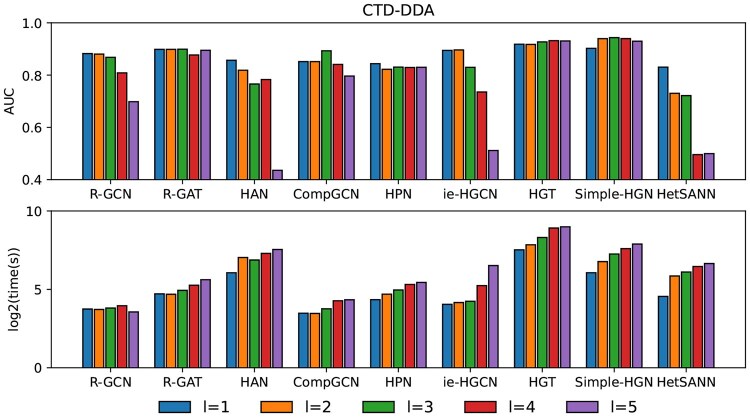
Performance and training time of different HGNNs in CTD-DDA under different GNN layers.

The GNN layer determines the depth of the model, and deeper networks can usually learn more complex graph structures, thereby improving the performance of the model. However, it may also lead to problems such as vanishing or exploding gradients. We evaluate the impact of the number of GNN layer *l* (from 1 to 5) on prediction performance and time efficiency of HGNNs. Figure 5 shows the prediction performance and time efficiency under different GNN layers for CTD-DDA dataset. It is found that in general the best number of layers is l≤3 as larger number of layers start to cause the model to over-smoothing. Meanwhile, as the number of layers increases, the complexity and computational cost of the model increase, thereby increasing the time cost (in seconds) of full training. We suggest that practitioners start exploring at a lower number of layers (i.e. l=1) of GNN to choose the appropriate model complexity and improve task performance. The influence of GNN layers on other datasets in Supplementary Section S8, available as supplementary data at *Bioinformatics Advances* online.

#### 4.3.3 Model specific hyperparameters

Further, we identify some key hyperparameters for the nine HGNN methods, which have also been identified within the literature as important. We carefully analyse and tune these hyperparameters by grid search, and compile our findings in Table 7. The table describes the various methods and their corresponding hyperparameters with some suggested guidelines.

**Table 7.** The general guidelines of recent HGNNs for setting model specific hyperparameters.

Methods	Hyperparameters	General guidelines
R-GCN	Hidden units: the number of units in hidden layer	Proper specification of hidden units value (e.g. 64) and layer value (e.g.l=2)
	l: the number of layers in neural network.	
R-GAT	Hidden units: the number of units in hidden layer	Proper specification of hidden units value (e.g. 64) and layer value (e.g.l=2); The number of attention heads is small (e.g. 3)
	l: the number of layers in neural network	
	Number of heads: multi-head attention mechanism.	
CompGCN	Hidden units: the number of units in hidden layer	Proper specification of hidden units value (e.g. 64) and layer value (e.g. 2 or 3); A good learning rate (e.g. η= 0.01)
	l: the number of layers in neural network	
	η : learning rate.	
HAN, HPN	*Meta-paths: guide nodes to randomly walk*	Pre-defined meta-paths (e.g. drug-target-drug) of the predicted link type (e.g. drug-target interaction)
HetSANN	l: the number of layers in neural network	A small number of layers (e.g. l=1); $\beta_{1}=10^{-4},\beta_{2}=10^{-5}$
	$\beta_{1},\beta_{2}$ : the weighting factors of cycle-consistency loss.	
ie-HGCN	l: the number of layers in neural network	The number of layers should change according to the structure of the graph (e.g. complex:l=3 or higher, simple:l=1 or 2);d=32
	d: the hidden layer dimensionality of type-level attention.	
HGT	Hidden units: the number of units in hidden layer	A large hidden units value (e.g. 256); A good number of attention heads (e.g. 8)
	Number of heads: multi-head attention mechanism.	
Simple-HGN	Hidden units: the number of units in hidden layer	A large hidden units value (e.g. 256); d=64
	d: edge-type embedding dimensionality.	

## 5 Conclusion

This article presents a benchmarking study, which provides an overview of various heterogeneous graph representation learning methods and evaluates their performance on the important biomedical task of link prediction. Specifically, we compile eight datasets from public databases and previous studies and use them to benchmark 42 representative HGNN methods, including nine general purpose HGNN methods. Through extensive experiments, we find that recent general purpose HGNN models can achieve comparable or even better performance compared with state-of-the-art techniques for biomedical link prediction tasks, and thus deserve more attention in future biomedical graph analysis both in practice and research. Further, HGNNs based on attention mechanisms are generally superior to the traditional convolutional computation mechanisms, more conducive to modeling high-order medical graphs, and more helpful for link prediction tasks. Finally, having analysed and tuned the important hyperparameters for HGNNs, we provide practitioners with general guidelines for tackling the challenge of setting hyperparameters in this domain.

Although this article focuses on link prediction tasks, it should be noted that node classification task is also crucial in the biomedical field. Our framework shown in Fig. 1 is extensible to tackle node classification tasks. Specifically, with the original graph containing the nodes whose categories are to be predicted, an HGNN is used as an encoder to embed the original graph, the final dimension of HGNN is set to the same as the number of node categories. For single-label classification tasks, *softmax* and cross entropy loss functions are commonly used. For multilabel datasets, *sigmoid* activation function and binary cross entropy loss function are commonly used (the interested reader is referred to our publicly available code: https://github.com/Zaiwen/Link_Prediction_in_Biomedical_Network for a demo on node classification tasks). In our future work, we will validate the practicality of our framework in real-world biomedical problems, such as cell function classification and identification of drug targets.

## Supplementary Material

vbaf187_Supplementary_Data

## Data Availability

All the code and data are available at https://github.com/Zaiwen/Link_Prediction_in_Biomedical_Network.
