# Supplementary material for: Heterogeneous graph neural networks for link prediction in biomedical networks
Source: Bioinform Adv. 2025 Aug 5;5(1):vbaf187. doi: 10.1093/bioadv/vbaf187 (PMC12448810; doi:10.1093/bioadv/vbaf187)
Supplement: vbaf187_Supplementary_Data [file vbaf187_supplementary_data.docx]

# Supplementary Materials

**Heterogeneous Graph Neural Networks for Link Prediction in Biomedical Networks**

Junwei Hu,^1^ Michael Bewong,^2,3^ Selasi Kwashie,^3^ Wen Zhang,^1,4,5^ Hong-Yu Zhang^1,4,5^ and Zaiwen Feng^1,4,5,∗^

^1^College of Informatics, Huazhong Agricultural University, Wuhan, Hubei, China, ^2^School of Computing, Mathematics & Engineering, Charles Sturt University, Wagga Wagga, NSW, Australia, ^3^Artificial Intelligence & Cyber Futures Institute, Charles Sturt University, Bathurst, NSW, Australia, ^4^Engineering Research Center of Agricultural Intelligent Technology, Ministry of Education and ^5^Hubei Key Laboratory of Agricultural Bioinformatics, Huazhong Agricultural University, Wuhan, Hubei, China

^∗^Corresponding author. E-mail: Zaiwen.Feng@mail.hzau.edu

**The Supplementary Materials file includes：**

**S1: Detailed description of heterogeneous graph neural networks**

**S2: Summary of biomedical network datasets**

**S3: Summary description and relevant source code links of baseline models**

**S4: Baseline methods and evaluation metrics *w.r.t.* network datasets**

**S5: Details of experiment settings**

**S6: Performance of recent HGNNs and baseline methods in ProGO-Net**

**S7: Performance of recent HGNNs and baseline methods in four networks**

**S8: Influence of hyper-parameters
S9: Running time of baseline techniques**

**S10: Performance of recent HGNNs on complex datasets**

**S1: Detailed description of heterogeneous graph neural networks**

- **R-GCN**: Relational graph convolutional network (R-GCN) is an extension of GCN designed for relational graphs (containing multiple edge types). It performs convolution by weighted summation of the outputs of multiple ordinary graph convolutions. Each convolution considers specific types of edges, enabling the model to capture richer information from the network structure. For each node *i*, the *l^th^* layer of convolution in R-GCN is defined as:

$h_{i}^{(l)}=\sigma\left(\sum_{r\in R} \sum_{j\in N_{i}^{r}} \frac{1}{c_{i,r}}W_{r}^{(l-1)}h_{j}^{(l-1)}+W_{0}^{(l-1)}h_{i}^{(l-1)}\right)$

where$h_{i}^{(l)}$is the representation of node *i* after *l^th^* layer, $N_{i}^{r}$represents the collection of indices for all neighboring nodes of node *i* corresponding to relation $r\in R$, and $c_{i,r}$ denotes a normalization constant, $\sigma$ is the activation function.

- **CompGCN**: CompGCN expands upon R-GCN by utilizing diverse entity-relation composition operations, thereby embedding nodes and relations jointly.
- **R-GAT**: Relational graph attention network (R-GAT) extends attention mechanisms to the relational graph through calculation weight $\alpha_{ij}^{r}$ for each edge $\langle i,j\rangle$:

$$\alpha_{ij}^{r}=\frac{\exp\left(\sigma\left(\text{a}^{T}\left[W_{r}h_{i}||W_{r}h_{j}\right]\right)\right)}{\sum_{r\in R} \sum_{k\in N_{i}^{r}} \exp\left(\sigma\left(\text{a}^{T}\left[W_{r}h_{i}||W_{r}h_{k}\right]\right)\right)}$$

where a denotes learnable weights, $W_{r}$represents the learnable parameters of a shared linear transformation, and || is the concatenation operator. Then, the neighborhood aggregation step as follows:

$$h_{i}^{(l)}=\sigma\left(\sum_{r\in R} \sum_{j\in N_{i}^{r}} \alpha_{ij}^{r}W_{r}h_{j}^{(l-1)}\right)$$

- **HAN**: Heterogeneous graph attention network (HAN) employs meta-paths instead of focusing on one-hop neighbors to capture higher-order proximity. For a given meta-path *M*, the representation of node *u* is derived from the aggregation of its neighbors based on meta-path, *i.e.*, *N_M_*(*u*) = {*uv* | *v* *connects with* *u via the meta-path M*}. HAN introduces an attention mechanism aimed at learning different weights to these neighbors, highlighting the more important ones for understanding node *u*:

$$\alpha_{uv}^{M}=\frac{\exp\left(\sigma\left(\text{a}_{M}^{T}\left[h_{u}^{'}||h_{v}^{'}\right]\right)\right)}{\sum_{k\in N_{M}(u)} \exp\left(\sigma\left(\text{a}_{M}^{T}\left[h_{u}^{'}||h_{k}^{'}\right]\right)\right)}$$

$$h_{u}^{M}=\sigma\left(\sum_{v\in N_{M}(u)} \alpha_{uv}^{M}h_{v}^{'}\right)$$

where $h_{u}^{'}$ and $h_{v}^{'}$ are the projected feature vectors of node *u* and *v* respectively, a*_M_* represents the node-level attention vector of the meta-path *M*. Based on the embedding $h_{u}^{M}$obtained from specific meta-path, HAN assigns weights to different meta-paths through the calculation of semantic-level attention:

$$\beta_{M}=\frac{\exp\left(\frac{1}{\left|V\right|}\sum_{u\in V} q^{T}\tanh\left(Wh_{u}^{M}+b\right)\right)}{\sum_{M'} \exp\left(\frac{1}{\left|V\right|}\sum_{u\in V} q^{T}\tanh\left(Wh_{u}^{M'}+b\right)\right)}$$

$$e_{u}=\sum_{M} \beta_{M}h_{u}^{M}$$

where *W* denotes the weight matrix, and *b* represents the bias vector, and *q* refers to the semantic-level attention vector, $e_{u}$is the final representation of node *u*.

- **HPN**: Heterogeneous graph propagation network (HPN) extends HAN model by incorporating two essential mechanisms: semantic propagation and semantic fusion. The former mechanism emphasizes the local semantics of each node during the process of aggregating from its neighbors and alleviates semantic confusion at the node level. Meanwhile, the latter mechanism learns the relative importance of different meta-paths for the specific task, and generates an optimally weighted combination of semantic-specific node embeddings.
- **HGT**: Inspired by the Transformer structure, Heterogeneous Graph Transformer (HGT) utilizes individual edge types to parameterize the attention mechanism resembling that of Transformer. In particular, HGT maps *v* into a *Query* vector and *u* into a *Key* vector for each edge $\langle u,v\rangle$, subsequently computing their dot product to derive attention:

$$Q_{v}^{i}=Q_{\varphi(v)}^{i}\left(h_{v}^{(l)}\right),K_{u}^{i}=K_{\varphi(u)}^{i}\left(h_{u}^{(l)}\right)$$

$$\text{Attention}_{HGT}\left(u,v\right)=\mathop{\text{softmax}}\limits_{u\in N(v)} \left(\mathop{||}\limits_{i\in[1,k]} ATT_{head}^{i}\left(u,v\right)\right)$$

where $h_{v}^{(l)}$ and $h_{u}^{(l)}$ are the output of the *l^th^* HGT layer, $Q_{\phi(v)}^{i}$ and $K_{\phi(u)}^{i}$ represent the node type-aware linear projection function, $ATT_{head}^{i}$ denote the *i^th^* attention head, $W_{\psi(u,v)}^{ATT}$ is an edge-based matrix, $\mu$ is a prior tensor representing adaptive scaling to the attention, and *k* represents the number of attention heads. In parallel with attention calculation, the message passing process can be computed as follows:

$$MSG_{head}^{i}\left(u,v\right)=M_{\varphi(u)}^{i}\left(h_{u}^{(l)}\right)W_{\psi(u,v)}^{MSG}$$

$$\text{Message}_{HGT}\left(u,v\right)=\mathop{||}\limits_{i\in[1,k]} MSG_{head}^{i}\left(u,v\right)$$

where $M_{\varphi(u)}^{i}$ and $W_{\psi(u,v)}^{MSG}$ are also the node type-aware linear projection function and matrix of incorporating edge dependency respectively. For aggregating the messages from the neighborhoods of node *v*, attention vectors are used as weights to obtain the updated vector:

$h'_{v}^{(l+1)}=\sum_{u\in N(v)} \text{Attention}_{HGT}\left(u,v\right)$⊙$\text{Message}_{HGT}\left(u,v\right)$

and further the output of the *l^th^* layer, according to the residual connection principle, is computed as follows:

$$h_{v}^{(l+1)}=A_{\varphi(v)}\left(\sigma\left(h'_{v}^{(l+1)}\right)\right)+h_{v}^{(l)}$$

where $A_{\varphi(v)}$ is a linear projection function that maps the vector of node *v* back to its node type-specific distribution.

- **HetSANN**: Heterogeneous Graph Structural Attention Neural Network (HetSANN), similar to HGT, utilizes an attention mechanism to analyze the importance of different types of nodes around a specific query node, enabling it to capture both interactions of inter-type nodes and assign different weights to neighbors during message aggregation.
- **Simple-HGN**: Lv *et al.* were inspired by the effectiveness of the straightforward GAT over more complex and specialized HGNNs, leading them to propose Simple-HGN. This approach extends the original graph attention mechanism by incorporating edge type information when calculating attention scores between nodes. To be specific, Simple-HGN allocates a *d*-dimensional embedding $h_{r}$ for each edge type $r=\psi(e)\in R$ at each layer. It then employs both edge type embeddings and node embeddings to compute the attention score:

$$\alpha_{ij}=\frac{\exp\left(\text{LeakyReLU}\left(\text{a}^{T}\left[Wh_{i}||Wh_{j}||W_{r}h_{\psi(\langle i,j\rangle)}\right]\right)\right)}{\sum_{r\in R} \mathop{\sum_{k\in N_{i}^{r}} \exp\left(\text{LeakyReLU}\left(\text{a}^{T}\left[Wh_{i}||Wh_{k}||W_{r}h_{\psi(\langle i,k\rangle)}\right]\right)\right)}\limits}$$

where $W_{r}$ represents a learnable matrix to transform type embeddings, and $\psi(\langle i,j\rangle)$ denotes the type of edge$\langle i,j\rangle$.

- **ie-HGCN**: Interpretable and Efficient Heterogeneous Graph Convolutional Network (ie-HGCN) is crafted to learn embeddings for heterogeneous graphs through a GCN that is specialized for node types. Initially, ie-HGCN maps the representations of diverse types of neighboring nodes into a unified semantic space. Then, in node-level aggregation, it treats the heterogeneous graph as a set of several bipartite graphs and applies GCN for bipartite graphs. Finally, ie-HGCN utilizes the attention mechanism to aggregate diverse types of neighboring nodes to generate node embeddings in type-level aggregation.

**S2: Summary of biomedical network datasets**

We utilize eight biomedical networks in this work, namely DeepViral-Net,ProGO-Net, NeoDTI-Net, deepDR-Net, CTD-DDA, NDFRT-DDA, DrugBank-DDI and STRING-PPI. Table S1 summarizes the node and edge statistics of eight network datasets.

| Table S1. Summary of biomedical network datasets | | | | |
| --- | --- | --- | --- | --- |
| Datasets | Type of nodes | Type of edges | Number of nodes | Number of edges |
| DeepViral-Net | HPO, GO, VS,  MGI, Protein | PHA, PGA, VMA,  VHA, PMA, PPI | 55197 | 803268 |
| ProGO-Net | GO, Protein | PGA, GGA, PPI | 34646 | 708173 |
| NeoDTI-Net | Drug, Target(Protein),  Disease, Side-effect | PDA, DSA, DDI,  DTI, DDA, PPI | 12015 | 1895445 |
| deepDR-Net | Drug, Target,  Disease, Side-effect | DSA, DDI,  DTI, DDA | 16677 | 686298 |
| CTD-DDA | Drug, Disease | DDA | 12765 | 92813 |
| NDFRT-DDA | Drug, Disease | DDA | 13545 | 56515 |
| DrugBank-DDI | Drug | DDI | 2191 | 242027 |
| STRING-PPI | Protein | PPI | 15131 | 359776 |

*Terminology: (1) Type of nodes: Human Phenotype Ontology (HPO); Gene Ontology (GO); virus species (VS); Mouse Genome Informatics (MGI). (2) Type of edges: Protein-HPO association (PHA); Protein-GO association (PGA); VS-MGI association (VMA); VS-HPO association (VHA); Protein-MGI association (PMA); Protein-Protein interaction (PPI); GO-GO association (GGA); Protein-Disease association (PDA); Drug-Side-effect association (DSA); Drug-Drug interaction (DDI); Drug-Target interaction (DTI); Drug-Disease association (DDA).*

**S3: Summary description and relevant source code links of baseline models**

We identified 33 state-of-the-art methods for benchmarking on the eight datasets across the four main link prediction tasks. Table S2 summarizes the information of 33 baseline models.

| Table S2. Summary description and relevant source code links of baseline models | | |
| --- | --- | --- |
| Model | Description | Link to the code |
| BioERP [M1] | Using two learning techniques to create a combined representation of biomedical entities and their connections | https://github.com/pengsl-lab/BioERP.git |
| DL2Vec [M2] | Analyzing gene-disease relationships by considering gene properties and their links to biomedical information | https://github.com/bio-ontology-research-group/DL2Vec |
| Onto2Vec [M3] | Learning features for biological entities based on how they are classified in biomedical ontologies | https://github.com/bio-ontology-research-group/onto2vec |
| OPA2Vec [M4] | Applying a pre-trained Word2Vec model to produce feature vectors of biological entities | https://github.com/bio-ontology-research-group/opa2vec |
| Node2Vec [M5] | Using a special random walk procedure to explore diverse neighborhoods | https://github.com/aditya-grover/node2vec |
| EL Embeddings  [M6] | A method for representing complex entities as simple vectors in a computer-usable space | https://github.com/bio-ontology-research-group/el-embeddings |
| TransE [M7] | A model that views relationships as translations between the vector representations of entities | https://github.com/pyg-team/pytorch\_geometric |
| SimResnik  [M8] | A similarity measure for evaluating semantic relatedness | https://github.com/bio-ontology-research-group/machine-learning-with-ontologies |
| SimLin [M9] | An information-theoretic definition of similarity |
| SiameseNN  [M10] | An architecture that is used for similarity learning |
| NeoDTI [M11] | Employing a network topology preservation step to ensure the learned drug and target representations align with the observed network | https://github.com/FangpingWan/NeoDTI |
| MSCMF [M12] | Leveraging multiple drug and target similarity matrices along with data-driven weighting to improve prediction accuracy and identify important interaction-related similarities | http://web.kuicr.kyoto-u.ac.jp/supp/yoshi/drugtarget/ |
| HNM [M13] | Using a heterogeneous network model to effectively identify drug repositioning by integrating disease, drug, and drug target data | http://cbc.case.edu |
| DTINet [M14] | Using a compact feature learning algorithm to generate low-dimensional vector representations that encode the topological features of each node in the network | https://github.com/luoyunan/DTINet |
| BLMNII [M15] | Integrating neighbor-based interaction-profile inferring into existing bipartite local models to handle drug-target prediction | https://github.com/lpeska/ALADIN/blob/master/ |
| DT-Hybrid  [M16] | Extending a well-known recommendation technique by incorporating drug and target similarity information | http://sites.google.com/site/ehybridalgo/ |
| NetLapRLS  [M17] | A manifold regularization semi-supervised learning method that combines labeled data with unlabeled data | https://github.com/stephenliu0423/PyDTI/blob/master/ |
| deepDR [M18] | Learning high-level features of each drug through a multi-mode deep autoencoder and analyzing known drug-disease relationships through a variational autoencoder | https://github.com/ChengF-Lab/deepDR |
| KBMF [M19] | Extending kernelized matrix factorization with a fully Bayesian framework and the ability to integrate information from multiple sources through different kernels | http://research.ics.aalto.fi/mi/software/kbmf/ |
| SVM [M20] | A machine learning approach for classifying data into two groups | https://scikit-learn.org/ |
| RF [M21] | A machine learning method that combines multiple decision tree to make predictions |
| RWR [M22] | Using diffusion state distance to measure the similarity of each pair of nodes | http://dsd.cs.tufts.edu/capdsd |
| Katz [M23] | Using functional connections between genes to predict disease associations | https://networkx.org/ |
| Laplacian  [M24] | A dimensionality reduction and data representation technique based on Laplacian Eigenmaps | https://github.com/thunlp/OpenNE |
| GF [M25] | A method partitions the network to minimize connections between machines for better efficiency |
| SVD [M26] | A mathematical technique for breaking down a matrix into simpler components | http://www.numpy.org/ |
| HOPE [M27] | A scalable graph embedding method to maintain high-order approximation of large scale graphs and capture asymmetric transitivity | https://github.com/ZW-ZHANG/HOPE |
| Grarep [M28] | An embedding method that combines the overall structure of the network to create representations of nodes | https://github.com/ShelsonCao/GraRep |
| DeepWalk  [M29] | An approach based random walk for learning latent representations of nodes | https://github.com/phanein/deepwalk |
| Struc2Vec  [M30] | Using a hierarchical approach to analyze similarities between nodes at different scales | https://github.com/ArnoldMeng/struc2vec |
| LINE [M31] | Using a specifically designed function and an edge-sampling algorithm to learn embeddings | https://github.com/tangjianpku/LINE |
| SDNE [M32] | Using a deep learning approach with multiple non-linear layers to capture the network's complex structure | https://github.com/suanrong/SDNE |
| GAE [M33] | An unsupervised learning method for graph-structured data based on a variational auto-encoder | https://github.com/tkipf/gae |

**S4: Baseline methods and evaluation metrics *w.r.t.* network datasets**

For each link prediction task, we identify key evaluation metrics from their original papers [M1, M3, M11, M18], as shown in Table S3.

| Table S3. Baseline methods and evaluation metrics *w.r.t.* network datasets | | |
| --- | --- | --- |
| Datasets | Baseline methods | Evaluation metrics |
| DeepViral-Net | [M1], [M2] | AUC, AUPR, ACC |
| ProGO-Net | [M3], [M4], [M5], [M6], [M7], [M8],  [M9], [M10] | Mean Rank(MR),  AUC, Hit@10/100 |
| NeoDTI-Net | [M1], [M11], [M12], [M13], [M14],  [M15], [M16], [M17] | AUC, AUPR |
| deepDR-Net | [M1], [M14], [M18], [M19], [M20],  [M21], [M22], [M23] | AUC, AUPR |
| CTD-DDA,  NDFRT-DDA,  DrugBank-DDI,  STRING-PPI | [M1], [M5], [M24], [M25], [M26], [M27],  [M28], [M29], [M30], [M31], [M32], [M33] | AUC, ACC, F1 score |

- Abbreviations: true negatives (TN), false negatives (FN), true positives (TP) and false positives (FP).
- AUC: the area under the ROC curve, whose x-axis represents the false positive rate ($\frac{\text{FP}}{\text{TN}+\text{FP}}$) and y-axis represents the true positive rate ($\frac{\text{TP}}{\text{TP}+\text{FN}}$).
- AUPR: the area under the PR curve, whose x-axis represents the recall ($\frac{\text{TP}}{\text{TP}+\text{FN}}$) and y-axis represents the precision ($\frac{\text{TP}}{\text{TP}+\text{FP}}$).
- ACC: the proportion of the predicted correct quantity to the total quantity, ACC = $\frac{\text{TP}+\text{TN}}{\text{TP}+\text{FP}+\text{FN}+\text{TN}}$
- Mean Rank (MR): the average of the sorted positions of all correct triples *i.e.* {head, relation, tail} denoted {h, r, t}. The smaller the value, the better.
- Hits@**N**: indicates how many correct triples *i.e.* {h, r, t} are finally sorted in top **N**, the larger the value, the better.
- F1 score: the harmonic mean of precision and recall, F1 score = $2\times\frac{\text{precision}\times\text{recall}}{\text{precision}+\text{recall}}$

**S5: Details of experiment settings**

For each dataset, the experimental set-up is as follows:

- **DeepViral-Net**: Following the guidelines in [M1], we utilize the leave-one-family-out (LOFO) cross-validation strategy that is designed to test how well the model performs when encountering a novel virus emerging from a novel virus family, where prior information regarding its protein interactions is lacking. We reserve one virus family (http://purl.obolibrary.org/obo/NCBITaxon

_11308) from the positive set for testing, while the rest of the families are used for training in LOFO cross-validation. Note that, the results are separated into the full dataset and high confidence dataset where we filter PPIs with a MIscore less than 0.4 in HPIDB. MIscore is a score that represents the degree of confidence in the existence of a particular interaction, 0.4 is the default value proposed in [M1].

- **ProGO-Net**: Following the guidance in [M3], the dataset randomly split known protein-protein pairs into an 80% training set and 20% testing set, using 20% of the training set as a validation set. We utilize a prediction function (used in TransE [M7]) to rank all pairs of proteins, and then consider the protein pairs that appear in the testing set of known interacting protein-protein pairs to evaluate the model by each evaluation metric (Hit@10/100, Mean Rank, AUC). Note that results are divided into Raw and Filtered categories in [M4]. Raw results consider all pairs of proteins, whereas Filtered results exclude pairs found in the training or validation sets.
- **NeoDTI-Net**: Following the guidelines in [M11], we adopt the ten-fold cross-validation test all positive pairs (known drug-target pairs) as well as randomly sampled negative pairs (unknown drug-target pairs). There are ten times more negative pairs than positive pairs. In each fold, a randomly chosen subset of 90% positive and negative pairs was used as training set, the remaining 10% positive and negative pairs were held out as the testing set. To reduce data bias, we conduct the ten-fold cross-validation procedure iteratively ten times and calculate the average value.
- **deepDR-Net**: Following the guidance in [M18], we employ the five-fold cross-validation test clinically reported drug-disease pairs. For each fold, a randomly chosen subset of 20% clinically reported drug-disease pairs, while an equivalent number of negative pairs are chosen randomly from unknown drug-disease pairs as the testing set to evaluate the model's performance, and the remaining 80% clinically reported drug-disease pairs with same number of randomly sampled negative/unknown pairs were used to train the model. Then, we repeat the five-fold cross-validation procedure ten times to compute the average value.
- **CTD-DDA; NDFRT-DDA; DrugBank-DDI; STRING-PPI**: For each of these, the testing set (20%) and training set (80%) are comprised of positive samples, and an equal number of negative samples randomly chosen from unknown interactions following the guidelines outlined in [M1].

For each technique, the default experimental hyper-parameters are as follows:

- **R-GCN**: learning rate = 0.01, weight decay = 0.0001, dropout = 0.2 , layers = 2, epochs = 200 for all datasets, hidden units = 64
- **CompGCN**: learning rate = 0.01, weight decay = 0.0001, dropout = 0.2 , hidden units = 32, epochs = 500 , layers = 2
- **R-GAT**: learning rate = 0.01, weight decay = 0.0005, dropout = 0.2 , attention heads = 3, hidden units =64, epochs = 350, layers = 2
- **HAN**: learning rate = 0.005, weight decay = 0.001, dropout = 0.6 , attention heads = 8, hidden units = 128, epochs = 200, layers = 1
- **HPN**: learning rate = 0.005, weight decay = 0.001, dropout = 0.6 , attention heads = 8, hidden units = 64, epochs = 200, layers = 2, alpha = 0.1
- **ie-HGCN**: learning rate = 0.001, weight decay = 0.0005, dropout = 0.2, epochs = 3500, hidden units = 64, dimension of attention vector = 32, layers = 3
- **HGT**: learning rate = 0.001, weight decay = 0.0001, dropout = 0.4, epochs = 500, hidden units = 64, attention heads = 8, layers = 2
- **HetSANN**: learning rate = 0.0001, weight decay = 0.0005, dropout = 0.2, epochs = 10000, hidden units = 128, attention heads = 16, layers = 1, negative slope used in the LeakyReLU = 0.2
- **Simple-HGN**: learning rate = 0.001, weight decay = 0.0005, dropout = 0.2, epochs = 500, hidden units = 256, attention heads = 8, negative slope used in the LeakyReLU = 0.05, dimension of edge-type embedding = 64, layers = 3

The meta-paths used in each dataset for methods HAN and HPN are as follows:

**DeepViral-Net**:*protein-VS-protein;protein-HPO-VS-HPO-protein;protein-MGI-VS-MGI-protein;VS-protein-VS;VS-HPO-protein-HPO-VS;VS-MGI-protein-MGI-VS;*

**ProGo-Net**:*protein-protein;protein-protein-protein;protein-GO-protein;protein-GO-GO-protein;*

**NeoDTI-Net**:*drug-drug;drug-protein-drug;drug-protein-protein-drug;drug-protein-disease-protein-drug;protein-protein;protein-drug-protein;protein-drug-drug-protein;protein-drug-disease-drug-protein;*

**deepDR-Net**:*drug-drug;drug-disease-drug;disease-drug-disease;disease-drug-drug-disease;*

**CTD-DDA,NDFRT-DDA**:*drug-disease-drug;disease-drug-disease;*

**DrugBank-DDI**:*drug-drug;drug-drug-drug;drug-drug-drug-drug;*

**STRING-PPI**:*protein-protein;protein-protein-protein;protein-protein-protein-protein;*

**S6: Performance of recent HGNNs and baseline methods in ProGO-Net**

The results on this dataset are divided into Raw and Filtered, as shown in Table S5. The best results are marked in **boldface**.

| Table S5. Performance of recent HGNNs and baseline methods in ProGO-Net | | | | |
| --- | --- | --- | --- | --- |
| Method | Raw | | | |
|  | Hits@10 | Hits@100 | Mean Rank(MR) | AUC |
| TransE | 0.05 | 0.24 | 3960.4 | 0.78 |
| SimResnik | 0.05 | 0.25 | 1933.6 | 0.88 |
| SimLin | 0.04 | 0.20 | 2287.9 | 0.86 |
| SiameseNN | 0.05 | 0.41 | 1881.1 | 0.90 |
| SiameseNN (Ont) | 0.05 | 0.38 | 1838.3 | 0.89 |
| EL Embeddings | 0.01 | 0.22 | 1679.7 | 0.90 |
| Onto2Vec | 0.05 | 0.24 | 2434.6 | 0.77 |
| OPA2Vec | 0.03 | 0.23 | 1809.7 | 0.86 |
| Random walk | 0.04 | 0.28 | 1942.6 | 0.85 |
| Node2Vec | 0.03 | 0.22 | 1860.5 | 0.86 |
| ie-HGCN | 0.05 | 0.36 | 1174.8 | 0.96 |
| HGT | 0.05 | 0.34 | 957.7 | 0.97 |
| Simple-HGN | **0.08** | **0.46** | **936.1** | **0.98** |
| Method | Filtered | | | |
|  | Hits@10 | Hits@100 | Mean Rank(MR) | AUC |
| TransE | 0.11 | 0.29 | 3890.6 | 0.79 |
| SimResnik | 0.09 | 0.30 | 1864.4 | 0.89 |
| SimLin | 0.08 | 0.23 | 2218.7 | 0.87 |
| SiameseNN | 0.15 | 0.64 | 1808.8 | 0.89 |
| SiameseNN (Ont) | 0.13 | 0.59 | 1766.3 | 0.89 |
| EL Embeddings | 0.02 | 0.26 | 1637.7 | 0.90 |
| Onto2Vec | 0.08 | 0.31 | 2391.2 | 0.77 |
| OPA2Vec | 0.07 | 0.26 | 1767.6 | 0.88 |
| Random walk | 0.10 | 0.34 | 1958.6 | 0.86 |
| Node2Vec | 0.07 | 0.28 | 1813.1 | 0.87 |
| ie-HGCN | 0.12 | 0.52 | 1109.7 | 0.96 |
| HGT | 0.10 | 0.49 | 892.0 | 0.97 |
| Simple-HGN | **0.18** | **0.66** | **870.7** | **0.98** |

**S7: Performance of recent HGNNs and baseline methods in four networks**

The results on four datasets (CTD-DDA,NDFRT-DDA,DrugBank-DDI,STRING-PPI) as shown in Table S6. The best results are marked in **boldface**.

| Table S6. Performance of recent HGNNs and baseline methods in four networks | | | | | | | | | | | | |
| --- | --- | --- | --- | --- | --- | --- | --- | --- | --- | --- | --- | --- |
| Method | CTD-DDA | | | NDFRT-DDA | | | DrugBank-DDI | | | STRING-PPI | | |
|  | AUC | ACC | F1 | AUC | ACC | F1 | AUC | ACC | F1 | AUC | ACC | F1 |
| BioERP | **0.966** | **0.906** | **0.907** | 0.971 | **0.938** | **0.939** | **0.930** | **0.853** | 0.858 | 0.926 | 0.856 | 0.858 |
| Laplician | 0.856 | 0.793 | 0.802 | 0.930 | 0.917 | 0.921 | 0.796 | 0.720 | 0.729 | 0.639 | 0.596 | 0.586 |
| SVD | 0.936 | 0.855 | 0.854 | 0.779 | 0.707 | 0.700 | 0.919 | 0.837 | 0.837 | 0.867 | 0.794 | 0.790 |
| GF | 0.884 | 0.808 | 0.805 | 0.720 | 0.660 | 0.655 | 0.882 | 0.720 | 0.802 | 0.810 | 0.746 | 0.747 |
| HOPE | 0.951 | 0.886 | 0.887 | 0.949 | 0.928 | 0.931 | 0.923 | 0.844 | 0.846 | 0.839 | 0.764 | 0.764 |
| GraRep | 0.960 | 0.899 | 0.900 | 0.963 | 0.931 | 0.934 | 0.925 | 0.845 | 0.846 | 0.894 | 0.823 | 0.822 |
| DeepWalk | 0.929 | 0.866 | 0.864 | 0.783 | 0.710 | 0.709 | 0.921 | 0.840 | 0.842 | 0.884 | 0.813 | 0.814 |
| Node2Vec | 0.911 | 0.838 | 0.835 | 0.819 | 0.742 | 0.741 | 0.902 | 0.819 | 0.819 | 0.828 | 0.758 | 0.756 |
| Struc2Vec | 0.965 | 0.903 | 0.903 | 0.958 | 0.913 | 0.921 | 0.904 | 0.826 | 0.830 | 0.909 | 0.838 | 0.841 |
| LINE | 0.965 | 0.904 | 0.904 | 0.962 | 0.934 | 0.935 | 0.905 | 0.825 | 0.829 | 0.859 | 0.788 | 0.795 |
| SDNE | 0.935 | 0.863 | 0.861 | 0.944 | 0.896 | 0.897 | 0.911 | 0.833 | 0.838 | 0.884 | 0.813 | 0.814 |
| GAE | 0.937 | 0.857 | 0.856 | 0.813 | 0.735 | 0.730 | 0.917 | 0.836 | 0.840 | 0.900 | 0.827 | 0.829 |
| ie-HGCN | 0.920 | 0.844 | 0.849 | 0.965 | 0.912 | 0.907 | 0.882 | 0.826 | 0.840 | 0.960 | 0.911 | 0.912 |
| HGT | 0.936 | 0.866 | 0.860 | 0.962 | 0.874 | 0.864 | 0.901 | 0.844 | 0.855 | 0.964 | 0.922 | 0.920 |
| Simple-HGN | 0.947 | 0.875 | 0.868 | **0.977** | 0.909 | 0.903 | 0.908 | 0.852 | **0.863** | **0.972** | **0.928** | **0.926** |

**S8: Influence of hyper-parameters**

We analyze the impact of different embedding dimensions and GNN layers on performance and training time in different datasets.


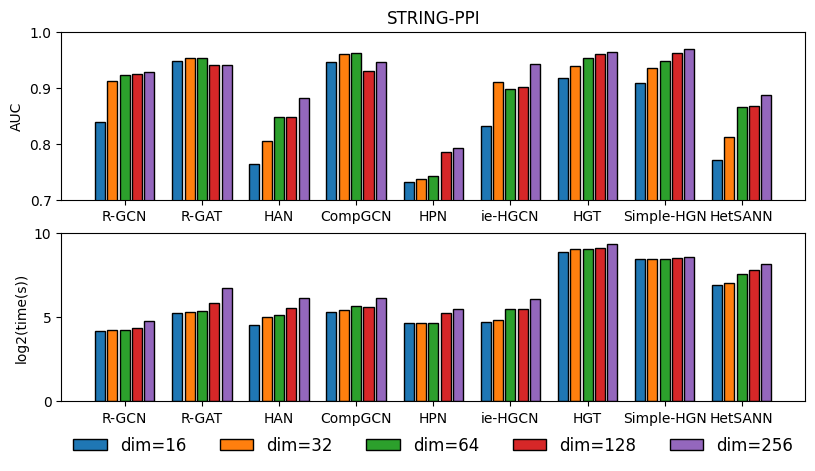


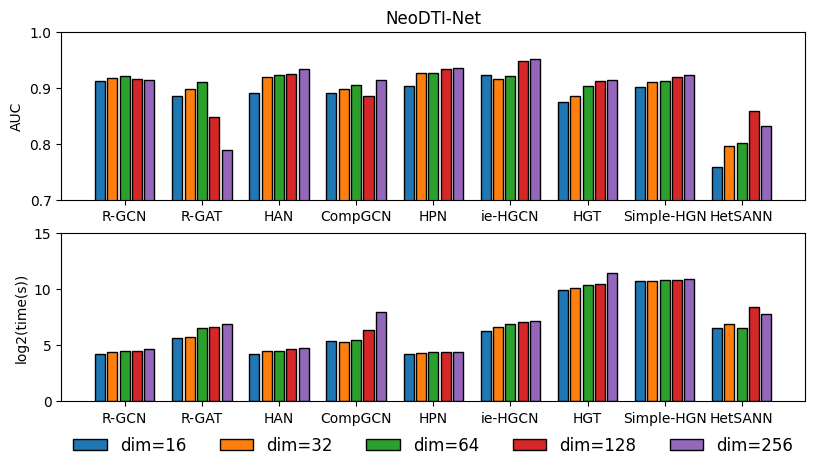


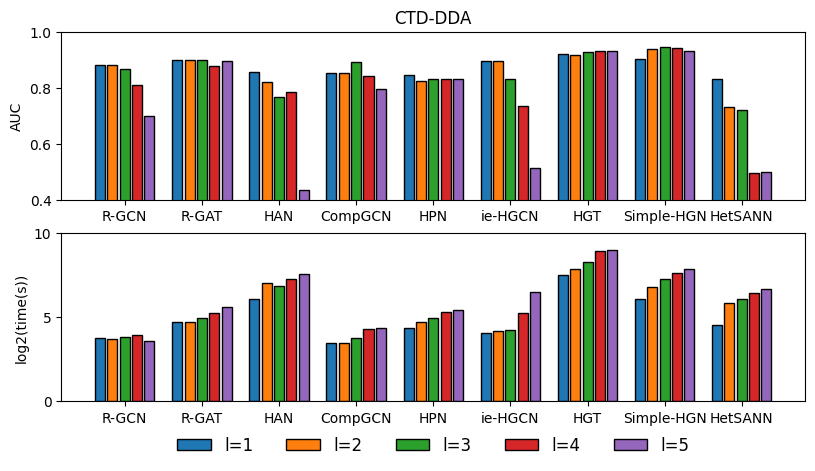


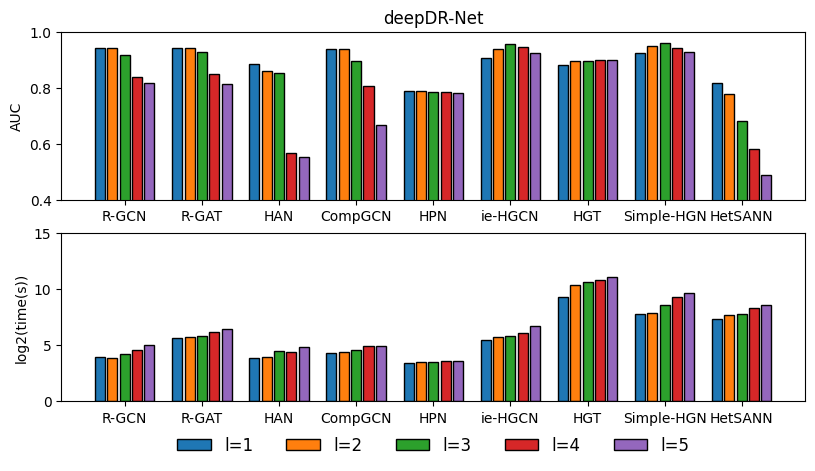


**S9: Running time of baseline techniques**

We calculated the full training time of end-to-end baseline models, as well as methods NeoDTI and deepDR designed specifically for biomedical networks, and compared them with general-purpose methods in terms of prediction performance and time efficiency in their respective datasets. Experimental results show that general-purpose methods achieve comparable and sometimes even better results while having a better time efficiency.


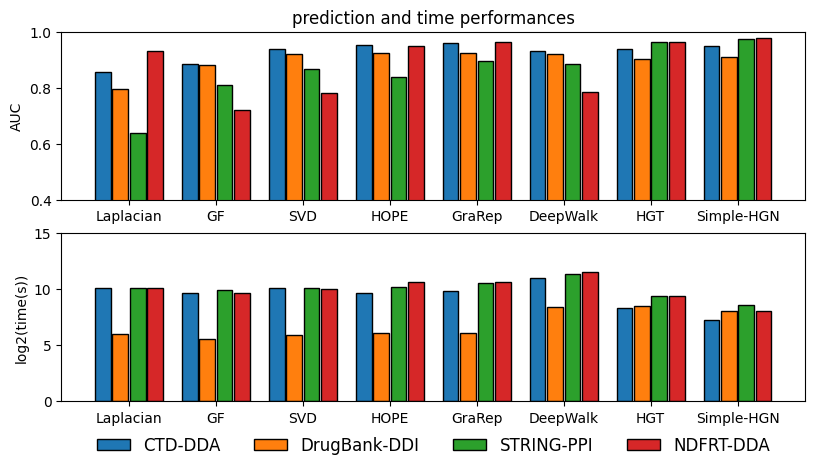


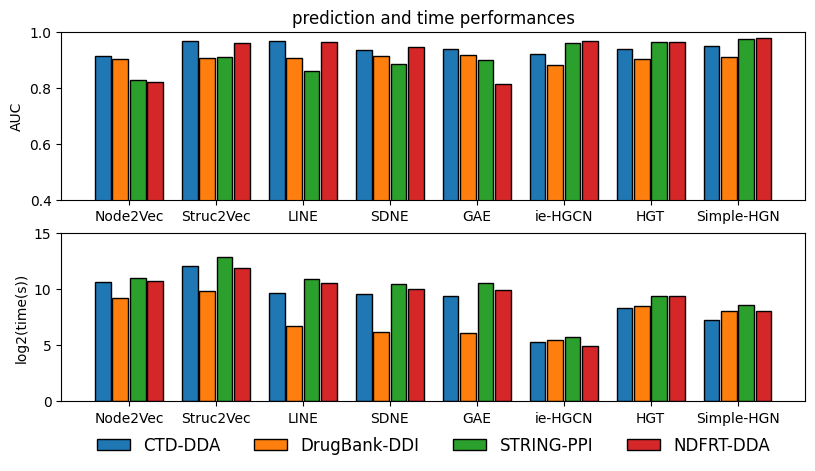


| Table S7. Performance of recent HGNNs and baseline methods in NeoDTI-Net and deepDR-Net | | | |
| --- | --- | --- | --- |
| Method | NeoDTI-Net | | |
|  | AUC | AUPR | log2(time(s)) |
| NeoDTI | 0.944 | 0.852 | 13.42 |
| HAN | 0.956 | 0.960 | 5.25 |
| HPN | 0.934 | 0.937 | **4.36** |
| Simple-HGN | **0.967** | **0.972** | 10.91 |
| Method | deepDR-Net | | |
|  | AUC | AUPR | log2(time(s)) |
| deepDR | 0.908 | 0.923 | 10.12 |
| R-GAT | 0.942 | 0.935 | 6.27 |
| ie-HGCN | 0.957 | 0.957 | **5.70** |
| Simple-HGN | **0.964** | **0.961** | 10.67 |

**S10: Performance of recent HGNNs on complex datasets**

In this section we identify two relatively complex link prediction biomedical network datasets, i.e., obgl-ppa and PubMed datasets. Obgl-ppa (https://ogb.standford.edu) is from the open graph benchmark relating to the biologically significant associations between proteins, which is used to train models to predict the association between proteins. PubMed (https://pubmed.ncbi.nlm.nih.gov) captures multiple biomolecules information and is often used to train models to predict the association between diseases. The detailed information of the data is shown in Table S8.

| Table S8. Summary of complex datasets | | | | |
| --- | --- | --- | --- | --- |
| Datasets | Type of nodes | Type of edges | Number of nodes | Number of edges |
| ogbl-ppa | 1 | 1 | 576289 | 30326273 |
| PubMed | 4 | 10 | 63109 | 244986 |

Then, the experimental results of applying different HGNNs to the two datasets are shown in Table S9. Overall, the results demonstrate their effectiveness. Compared with the PubMed dataset, the obgl-ppa network is larger in scale and simpler in structure, so the more complex HGNN is not as good as the simpler HGNN in performance, that is, R-GCN and R-GAT achieved better performance in AUC and AUPR evaluation metrics. In the PubMed data, due to the smaller scale and more complex structure, the complex HGNN works better, that is, Simple-HGN and HGT achieved better performance.

| Table S9. Performance of recent HGNNs on complex datasets | | | |
| --- | --- | --- | --- |
| Method | ogbl-ppa | | |
|  | AUC | AUPR | log2(time(s)) |
| R-GCN | **0.820** | 0.801 | 9.95 |
| R-GAT | **0.820** | **0.806** | 10.92 |
| CompGCN | 0.766 | 0.697 | 11.21 |
| ie-HGCN | 0.670 | 0.611 | **9.60** |
| HAN | 0.651 | 0.612 | 10.22 |
| HPN | 0.678 | 0.688 | 10.10 |
| HGT | 0.724 | 0.742 | 11.29 |
| HetSANN | 0.577 | 0.641 | 10.98 |
| Simple-HGN | 0.782 | 0.766 | 11.39 |
| Method | PubMed | | |
|  | AUC | AUPR | log2(time(s)) |
| R-GCN | 0.811 | 0.768 | 6.70 |
| R-GAT | 0.711 | 0.732 | 7.71 |
| CompGCN | 0.825 | 0.847 | **5.38** |
| ie-HGCN | 0.828 | 0.822 | 6.41 |
| HAN | 0.662 | 0.634 | 6.01 |
| HPN | 0.693 | 0.731 | 6.00 |
| HGT | 0.847 | 0.873 | 7.79 |
| HetSANN | 0.724 | 0.724 | 7.41 |
| Simple-HGN | **0.854** | **0.872** | 7.48 |

The additional experimental results are consistent with our findings that the generic HGNNs can be effective in link prediction tasks, even for the more complex datasets. It is worth noting that, although the results for the complex datasets are comparable to the results in existing published works such as in the work of Lv et al.[[1]](#footnote-0), the results show that, the AUC and AUPR for complex datasets are somewhat less than those of the other datasets. This indicates the impact of the complexity of a dataset on the effectiveness of link prediction techniques.

1. Lv, Q., Ding, M., Liu, Q., Chen, Y., Feng, W., He, S., ... & Tang, J. (2021, August). Are we really making much progress? revisiting, benchmarking and refining heterogeneous graph neural networks. In Proceedings of the 27th ACM SIGKDD conference on knowledge discovery & data mining (pp. 1150-1160). [↑](#footnote-ref-0)
